# ﻿Caribbean Amphipoda (Crustacea) of Panama. Part IV: parvorder Caprellidira

**DOI:** 10.3897/zookeys.1234.145826

**Published:** 2025-04-10

**Authors:** Sally J. Sir, Kristine N. White

**Affiliations:** 1 Aquatic Sciences Center, Department of Biological and Environmental Sciences, Georgia College & State University, Milledgeville, GA 31061, USA Georgia College & State University Milledgeville United States of America

**Keywords:** Bocas del Toro, Caprellidae, Caprellidira, identification key, Ischyroceridae, Neomegamphopidae, Photidae, Podoceridae

## Abstract

The parvorder Caprellidira includes 1,244 described species in 17 families. The diverse morphology of caprellidiran amphipods ranges from thread-like to more typical laterally compressed body forms. Caprellidiran amphipods are associated with coral rubble, seagrasses, sponges, algae, and sand and typically feed on detritus from the water column. Twenty species from five families within the parvorder are documented from Bocas del Toro, Panama. Five species are new to science and a range extension is documented for 15 species. All species are diagnosed, new species are described, and an identification key to the Caprellidira amphipods of Panama is provided herein.

## Abstract (es)

El parvorden Caprellidira incluye 1,244 especies descritas, distribuidas en 17 familias, con una morfología que varía desde formas corporales filiformes hasta las típicamente comprimidas lateralmente. Estas especies están asociadas a hábitats como escombros de coral, pastos marinos, esponjas, algas y sedimentos arenosos, donde suelen alimentarse de detritos en la columna de agua. En Bocas del Toro, Panamá, se han registrado 20 especies pertenecientes a cinco familias, de las cuales cinco representan descubrimientos nuevos para la ciencia, y se ha documentado una expansión del rango de distribución para 15 especies. Se diagnostican todas las especies, se describen nuevas especies y se proporciona una clave de identificación de los anfípodos Caprellidiran de Panamá.

## ﻿Introduction

Caprellidira Leach, 1814 (sensu Lowry and Myers 2013) is a parvorder consisting of 1,224 species distributed in a cosmopolitan manner (Horton et al. 2024). The parvorder Caprellidira was originally classified as infraorder Caprellida (Myers and Lowry 2003) based on the hypothesis that ancestors of these species consumed suspended matter found in the water column. This feeding behavior is reflected in several families such as Caprellidae which hold on to substrate with pereopods 5–7 and catch suspended matter as it drifts by. Amphipods in the genus *Cerapus* have a thick article 1 on antenna 1, which may provide extra strength to collect suspended matter with their antennae. Morphology varies drastically in the Caprellidira, with members of Caprellidae exhibiting threadlike bodies and other families with laterally compressed or subcylindrical bodies. Caprellida was reclassified as the parvorder Caprellidira by Lowry and Myers (2013).

The Caprellidira comprises 17 families: Aetiopedesidae Myers & Lowry, 2003 (one sp.); Australomicroprotopidae Myers, Lowry & Billingham, 2016 (one sp.); CaprellidaeLeach 1814 (452 spp.); Caprogammaridae Kudrjaschov & Vassilenko, 1966 (two spp.); Cyamidae Rafinesque, 1815 (29 spp.); Dulichiidae Dana, 1849 (30 spp.); Isaeidae Dana, 1852 (seven spp.); Ischyroceridae Stebbing, 1899 (293 spp.); Kamakidae Myers & Lowry, 2003 (43 spp.); Microprotopidae Myers & Lowry, 2003 (five spp.); Neomegamphopidae Myers, 1981 (22 spp.); Paragammaropsidae Myers & Lowry, 2003 (two spp.); Photidae Boeck, 1871 (238 spp.); Podoceridae Leach, 1814 (93 spp.); Priscomilitaridae Hirayama, 1988 (three spp.); Protodulichiidae Ariyama, 2019, in Ariyama and Hoshino 2019 (one sp.); Rakiroidae Myers & Lowry, 2003 (one sp.).

Prior to this study, 70 caprellidiran species in six families were documented from Caribbean waters: Caprellidae, Ischyroceridae, Kamakidae, Neomegamphopidae, Photidae, and Podoceridae (LeCroy et al. 2009; Miloslavich et al. 2010; Martín et al. 2013). *Paracaprellabarnardi* (McCain, 1967) is the only species previously documented from Caribbean Panama and *Posophotisseri* Barnard, 1979 was previously documented from the canal zone on the Pacific side of Panama (Barnard 1979). Twenty Caprellidira species were collected during this study, including five species new to science.

## ﻿Materials and methods

Coral rubble, algae, sand, seagrass, hydroids, sponges, and buoy scrapings were collected at 13 sites around Bocas del Toro, Panama at depths of 0.2–15 m. Substrates were elutriated with freshwater and amphipods were sorted into morphospecies while alive. Live specimens were placed in clove oil for imaging and preserved in 99.5% EtOH. Preserved specimens were examined in glycerol after being measured from the tip of the rostrum to the base of the telson. Amphipods were dissected using a stereomicroscope and illustrated using an Olympus BH2 differential interference contrast microscope with an Olympus BH2-DA drawing tube attached. Pencil drawings were digitally inked using a Wacom^®^ Intuos Pro Pen tablet following the methods of Coleman (2003) in Adobe Illustrator 2020. Abbreviations used in figures are as follows: **H**, habitus; **Hd**, head; **A**, antenna; **Mx**, maxilla; **Md**, mandible; **UL**, upper lip; **LL**, lower lip; **Xpd**, maxilliped; **C**, coxa; **G**, gnathopod; **P**, pereopod; **E**, epimeron; **Pl**, pleopod; **U**, uropod; **T**, telson; **R**, right; **L**, left. Size ranges of each species collected from Bocas del Toro, Panama are provided at the beginning of each material examined section. Specimens are deposited in the Smithsonian Institution, U.S. National Museum of Natural History (**USNM**) and the Gulf Coast Research Laboratory Museum (**GCRL**).

## ﻿Results

### ﻿Taxonomic account

**Parvorder Caprellidira Leach, 1814 (sensu Lowry & Myers, 2013)**

**Superfamily Caprelloidea Leach, 1814**

**Family Caprellidae Leach, 1814**

#### ﻿Genus Deutella

Taxon classificationAnimaliaAmphipodaCaprellidae

Mayer, 1890

B0D5758F-5E5B-593B-8C9E-EC9968EB0A09

##### Diagnosis.

Antenna 2 flagellum bi-articulate, lacking swimming setae. Mandibular palp tri-articulate. Maxilliped inner lobe shorter than outer lobe. Pereopod 5 with six articles, distinctly thinner than pereopods 6 and 7. Male abdomen with two appendages.

#### ﻿ Deutella caribensis

Taxon classificationAnimaliaAmphipodaCaprellidae

Guerra-García, Krapp-Schickel & Müller, 2006

3E2337B2-5DFC-5D3C-BDA0-32CBBE410999

Figs 1 , 31A

Deutella caribensis Guerra-García, Krapp-Schickel & Müller, 2006: 161–164, figs 7, 8.

##### Material examined.

Panama • 3.5 mm • 1 ♀; Bocas del Toro, Bocas del Drago; 9.4134°N, 82.3334°W; depth 1–3 m, among coral rubble, 23 June 2023; K.N. White leg.; USNM 1743942.

##### Diagnosis.

Head with paired dorsal projections. Body without lateral projections; pereonites 2–4 with dorsal projections. Pereopods 3 and 4 uni-articulate. Pereopod 5 longer than pereonite 5.

##### Distribution.

Colombia: Bahía Concha (Guerra-García et al. 2006); Panama: Bocas del Toro (present study).

##### Ecology and remarks.

This species occurs among algae and coral rubble at depths of 1–3 m. Panamanian specimens agree closely with the original description of this species. This is the first record of this species since the original description suggesting that the range of this species is much larger than previously known. Live specimens are yellow-brown in color with a red eye.

**Figure 1.**
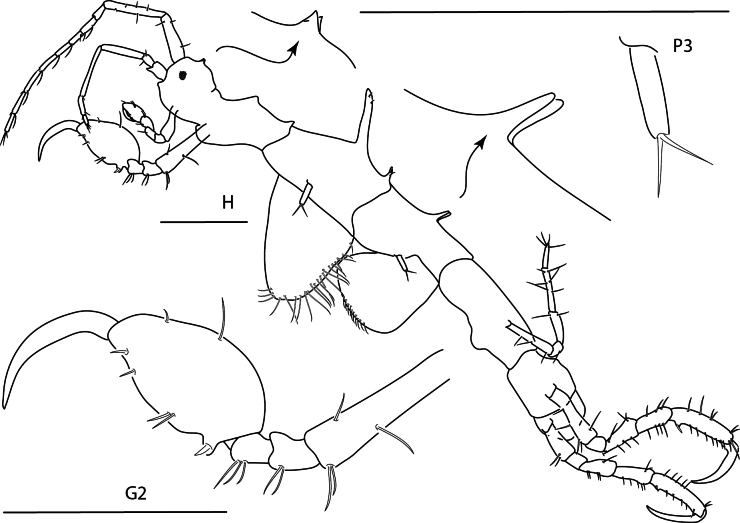
*Deutellacaribensis*, female, 3.5 mm, habitus, gnathopod 2 lateral, and pereopod 3. Scale bars: 0.5 mm.

#### ﻿ Deutella cf. pseudoincerta

Taxon classificationAnimaliaAmphipodaCaprellidae

Winfield & Guerra-García, 2021

A1279FC9-6832-5A8F-9807-84BD4D142DBD

Figs 2 , 31B

Deutella pseudoincerta Winfield & Guerra-García, 2021: 4–8, figs 2–6.

##### Material examined.

Panama • 2.2–2.8 mm • 3 ♂, 1 ♀; Bocas del Toro, Crawl Caye; 9.2459°N, 82.1369°W; depth 1–4 m, among coral rubble, 25 June 2023; K.N. White leg.; USNM 174393.

##### Diagnosis.

Head and pereonites 2 and 3 with dorsal projections; pereonite 4 with posterodorsal hump; pereonites with minute setae. Male gnathopod 2 propodus with distinct excavation and single grasping spine proximally. Pereopods 3 and 4 minute, bi-articulate, ~ 0.3 × length of gills. Pereopod 5 dactylus reduced to minute article. Male abdomen with two setose lobes and two setose appendages.

##### Distribution.

Mexico: Veracruz State (Winfield and Guerra-García 2021); Panama: Bocas del Toro (present study).

##### Ecology and remarks.

This species occurs among coral rubble at depths of 1–4 m. Panamanian specimens agree closely with previous descriptions of the species except for having five flagellar segments on antenna 1 (8 in original description) and more apically rounded pereopods 3 and 4 (triangular in original description). The size of pereopod 5 in illustrated specimen seems to be an anomaly, as all other specimens were missing pereopod 5. Variation among specimens collected in Panama includes differences in dorsal projections on body and proximal projections on gnathopod 2 and pereopods 6 and 7. Due to the morphological variation in specimens, comparison with type material is necessary to confirm the species identification. Live specimens are white in color with purple-brown splotches and a red eye.

**Figure 2.**
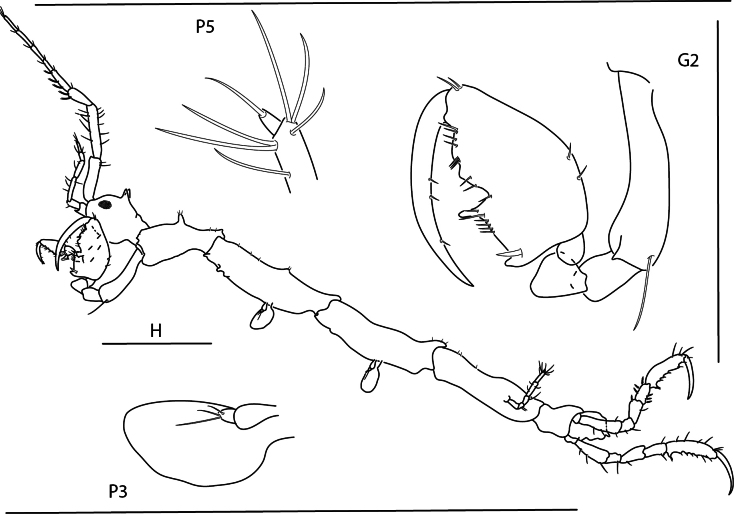
Deutellacf.pseudoincerta, male, 2.8 mm, habitus, gnathopod 2 medial, pereopod 3, and pereopod 5 dactylus. Scale bars: 0.5 mm.

#### ﻿Genus Paracaprella

Taxon classificationAnimaliaAmphipodaCaprellidae

Mayer, 1890

8ABE3C67-6C9D-5DDF-A2CC-D0B67BA1F407

##### Diagnosis.

Pereopods 3 and 4 bi-articulate. Maxilliped outer lobes significantly larger than inner lobes, not fused, bearing few setae. Mandibular palp reduced.

#### ﻿ Paracaprella pusilla

Taxon classificationAnimaliaAmphipodaCaprellidae

Mayer, 1890

6C73BB88-7256-582C-B0BB-F1FCCAD01D03

Figs 3 , 31C

Caprella nigra : Reid 1951: 283–284, 289, fig. 58.
Paracaprella pusilla Mayer, 1890: 41, taf. 1, figs 28–30, taf. 3, figs 45–47, taf. 5, fig. 48–49, taf. 6, fig. 10; Mayer 1903: 67, taf. 2, figs 36, 37, taf 7, fig. 52; Steinberg and Dougherty 1957: 283–284, figs 16, 19, 24, 30; McCain 1968: 82–86, figs 41, 42; Wakabara et al. 1991: 73; Guerra-García et al. 2006: 175, figs 17–19; Díaz et al. 2005: 6, 7, 22, fig. 13; Ros and Guerra-García 2012: 137; Ros et al. 2013: 677, fig. 2.

##### Material examined.

Panama • 3.2–3.8 mm • 2 ♂, 2 ♀; Bocas del Toro, Hospital Point; 9.3333°N, 82.2185°W; depth 11 m; from buoy scrapings, 26 June 2023; K.N. White leg; USNM 1743944.

##### Diagnosis.

Body lacking dorsal projections. Male pereonite 2 with large, triangular anteroventral projection. Gnathopod 1 dactylus reaching ~ 1/2 of propodus length. Male gnathopod 2 basis with posteroproximal bump.

##### Distribution.

Africa: West Africa (Reid 1951); Brazil: Rio de Janerio (Wakabara et al. 1991); Chile: Coquimbo (Guerra-García and Thiel 2001); Colombia: Magdalena (Guerra-García et al. 2006); Mediterranean Sea: Balearic Islands (Ros et al. 2013); Mexico: Gulf of Mexico (Steinberg and Dougherty 1957; Winfield et al. 2006); U.S.A.: Florida (Camp 1998); Venezuela: Falcón, Carabobo, Aragua, Sucre, Nueva Esparta (Díaz et al. 2005); Western North Atlantic (McCain 1968); Spain: Cadiz (Ros and Guerra-García 2012); Panama: Bocas del Toro (present study).

##### Ecology and remarks.

This species occurs among mangrove roots, seagrasses, hydroids, ascidians, gravel bottoms, ropes, mussels, oysters, and shallow waters (McCain 1968; Díaz et al. 2005; Guerra-García et al. 2006). In Bocas del Toro, this species was collected from buoy scrapings at 11 m depth. Panamanian specimens agree closely with the original description of the species; however, several descriptions show variation in the shape of the male gnathopod 2 propodus. This species has recently been documented as spreading in several non-indigenous regions (Ros and Guerra-García 2012; Ros et al. 2013). Live specimens are yellow-brown in color with brown spots and a brown eye.

**Figure 3.**
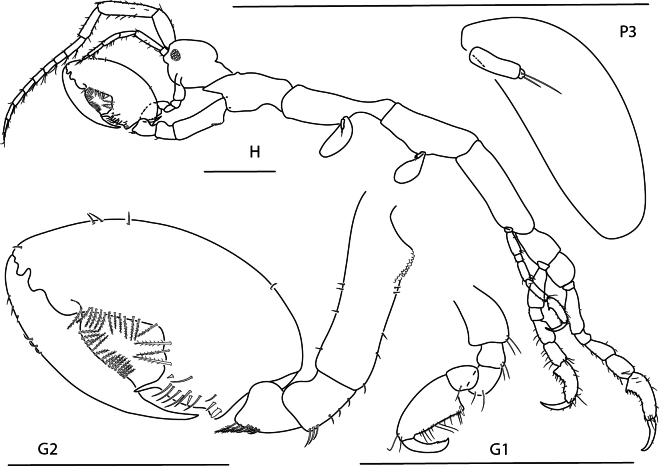
*Paracaprellapusilla*, male, 3.8 mm, habitus, gnathopod 2 lateral, gnathopod 1 lateral, and pereopod 3. Scale bars: 0.5 mm.

#### ﻿Family Ischyroceridae Stebbing, 1899

##### ﻿Genus Caribboecetes

Taxon classificationAnimaliaAmphipodaIschyroceridae

Just, 1983

147C5BDC-F8A9-5310-BD5D-3B774302ED5F

###### Diagnosis.

Body subcylindrical. Rostrum pointed. Gnathopods 1 and 2 simple. Coxae 3 and 4 distal margins dentate, setose. Pereopods 5–7 lacking accessory tooth. Urosomite 3 fused to telson. Uropod 1 biramous, inner ramus shorter than outer ramus. Uropod 2 absent. Uropod 3 rami absent.

##### ﻿ Caribboecetes intermedius

Taxon classificationAnimaliaAmphipodaIschyroceridae

Just, 1984

07EE5959-E4B6-5C1F-8320-214C0CFFF808

Figs 4 , 31D

Caribboecetes intermedius Just, 1984: 48, 49, figs 9, 10.
Caribboecetes sp.: Ortiz and Lemaitre 1994: 124.
Caribboecetes justi : Ortiz and Lemaitre 1997: 82–85, figs 10–14.

###### Material examined.

Panama • 1.1–4 mm • 7 ♂, 11 ♀, 12 juveniles; Bocas del Toro, Crawl Caye, 9.2449°N, 82.1383°W; depth 1.5–2.4 m, in sand; 11 Aug 2021; K.N. White leg.; USNM 1743945 • 3 ♂, 3 ♀, 7 juveniles; Bocas del Toro, Crawl Caye; 9.2475°N, 82.1290°W; depth 4.6 m, in sand; 12 Aug 2021; K.N. White leg.; USNM 1743946 • 1 ♀; Bocas del Toro, Bocas del Drago; 9.4134°N, 82.3334°W; depth 1–3 m, in sand; 23 June 2023; K.N. White leg.; USNM 1743947 • 3 ♂, 7 ♀; Bocas del Toro, Drago Beach; 9.4171°N, 82.3248°W; 0–1 m, in sand; 27 June 2023; K.N. White leg.; USNM 1743948.

###### Diagnosis.

Rostrum acute, reaching beyond eye lobes. Coxae 1–4 ventral margin with long setae; coxa 2 with plumose setae; coxae 3 and 4 anterodistal margins subtruncate. Gnathopod 2 propodus posterior margin with 1–3 robust setae. Pereopod 7 anterior and posterior margins with long, plumose setae.

###### Distribution.

Barbados: Bath (Just 1984); Colombia: Barú, Islas del Rosario, Gulf of Morrosquillo (Ortiz and Lemaitre 1997); Panama: Bocas del Toro (present study).

###### Ecology and remarks.

*Caribboecetesintermedius* is a tube dwelling species that occurs in sand at depths of 1–3 m. Gnathopod 2, propodus posterior margin with robust setae (number of robust setae varying with size). After observing the type specimens for *Caribboecetesjusti* and *Caribboecetesintermedius*, we believe that they are the same species. Panamanian specimens agree closely with previous descriptions of both species. Live specimens are yellow-white in color with brown markings on head and antennae.

**Figure 4.**
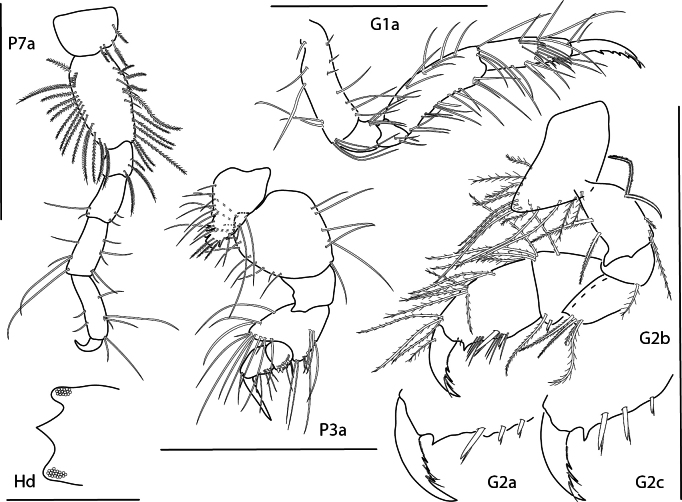
*Caribboecetesintermedius*, male “a” 3.0 mm, pereopod 7, pereopod 3, gnathopod 1 lateral, gnathopod 2 medial; female 2.4 mm, head; male “b” 1.4 mm, gnathopod 2 propodus and dactylus; male “c” 2.1 mm, gnathopod 2 propodus and dactylus. Scale bars: 0.5 mm.

##### ﻿Genus Cerapus

Taxon classificationAnimaliaAmphipodaIschyroceridae

Say, 1817

024E484C-A6DF-5D2A-81CE-C23E461EF552

###### Diagnosis.

Body subcylindrical. Rostrum produced. Antenna 1 peduncle article 1 expanded, wider than articles 2–3. Mandibular palp reaching beyond incisor process, tri-articulate. Coxae 1–4 discontinuous. Gnathopod 1 subchelate. Male gnathopod 2 carpochelate. Female gnathopod 2 subchelate, lacking stout setae. Pereopod 5 geniculate at merus; merus posteroventral margin produced. Pereopod 7 longer than pereopod 6. Pleopods 2 and 3 inner ramus shorter than outer. Uropod 1 inner ramus shorter than outer ramus. Uropods 2 and 3 uniramous. Telson cleft.

##### ﻿ Cerapus benthophilus

Taxon classificationAnimaliaAmphipodaIschyroceridae

Thomas & Heard, 1979

5F625EFE-1127-5DED-BB2B-851EE7E6B716

Figs 5 , 31E

Cerapus sp.: Thomas 1976: 92, 93.
Cerapus benthophilus Thomas & Heard, 1979: 98–104, figs 1–4; LeCroy 2007: 552, fig. 475.

###### Material examined.

Panama • 5.4 mm • 1 ♀; Bocas del Toro, Chiriqui Grande, Laguna de Chiriqui; 8.9396°N, 82.1105°W; depth 0.2–1.5 m, among *Thalassia*; 10 Aug 2005; S. LeCroy leg.; GCRL 6661.

###### Diagnosis.

Head ocular lobe posteriorly upturned; rostrum slightly produced. Antenna 1 flagellum 6-articulate. Pleopod 2 outer ramus unsegmented; inner ramus with marginal plumose setae. Uropod 1 outer ramus wide, apical margin not narrowing distally; inner ramus with apical robust seta, distal margin of seta narrowing unevenly.

###### Distribution.

U.S.A.: Ocean Springs, Mississippi (Thomas and Heard 1979), Indian River Lagoon, St. Lucie River, Biscayne Bay, southeastern Gulf of Mexico between Cape Sable and Cape Romano, Estero Bay and Cocohatchee River, Withalacoochee Bay, Florida panhandle to Louisiana, Florida (Nelson 1995; Thomas 1993; LeCroy 2007); Mexico: Laguna de Alvarado, Veracruz (Winfield et al. 1997, 2001), Laguna de Términos, Campeche (Ledoyer 1986); Panama: Chiriqui Grande, Laguna de Chiriqui (present study).

###### Ecology and remarks.

This species occurs among *Thalassia* at depths of 0.2–1.5 m. This species can be difficult to identify with only female specimens. Previously, male specimens have been identified based on the following characteristics: coxa 3 with small anterior lobe, reaching body lengths longer than most *Cerapus* species, ranging from 4–13 mm, male pereon segment 1 with lateral keel, male gnathopod 2 basis, anterodorsal margin with numerous, long setae, antennae 1 and 2, 7–12 segmented (LeCroy 2007; Thomas and Heard 1979). The number of antennae segments seems variable based on size, as seen with the specimens described herein and by Drumm (2018). Panamanian specimens agree closely with the original description of *Cerapusbenthophilus* Thomas & Heard, 1979. Ethanol-preserved specimens retained purple coloration on most of the body, especially on the head.

**Figure 5.**
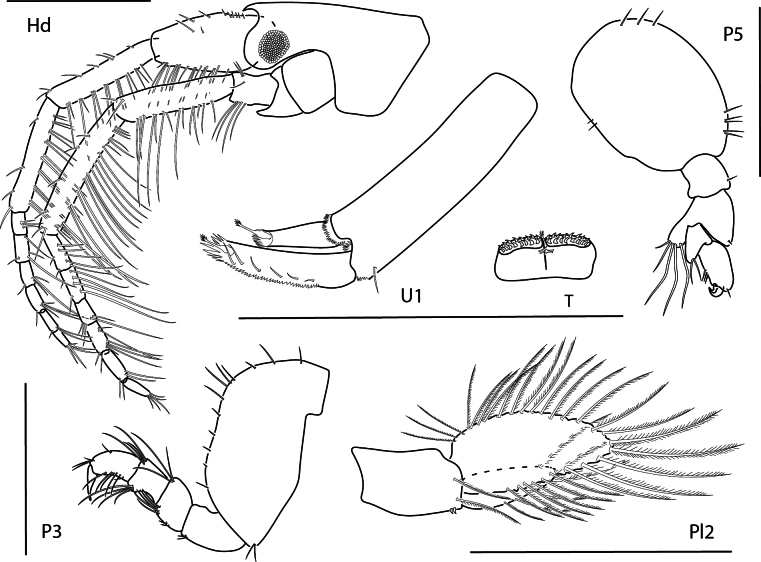
*Cerapusbenthophilus*, female, 5.4 mm, head, uropod 1, telson, pereopod 5, pereopod 3, and pleopod 2. Scale bars: 0.5 mm.

##### ﻿ Cerapus slayeri

Taxon classificationAnimaliaAmphipodaIschyroceridae

Drumm, 2018

D68D4BB0-F21B-5D65-BE4E-532624F3EC15

Figs 6 , 31F

Cerapus sp B.: LeCroy 2007: 556, fig. 481.
Cerapus slayeri Drumm, 2018: 496–503, figs 1–6.

###### Material examined.

Panama • 1.4–2 mm • 3 juvenile ♀; Bocas del Toro, Pidgeon Key Reef; 9.2693°N, 82.2489°W, depth 0.5–1 m, among *Halimeda*, *Thalassia*; 9 August 2005; S. LeCroy leg.; GCRL 6662.

###### Diagnosis.

Head ocular lobe posterior margin even, reaching ~ 1/2 of head length; rostrum short, acute. Antennae 1 and 2 flagella tri-articulate. Pereopod 7 basis posterior margin with spinules; carpus antero- and posterodistal margins with long, plumose setae. Pleopod 2 outer ramus tri-articulate; inner ramus with marginal setules. Uropod 1 inner ramus with apical robust seta, distal margin of seta narrowing evenly; both rami with marginal setules.

###### Distribution.

USA: Delaware Bay, Delaware and Great South Bay, New York (Drumm 2018); Florida (LeCroy 2007); Panama: Bocas del Toro (present study).

###### Ecology and remarks.

This species occurs among *Halimeda*, *Thalassia*, and mangrove roots at depths of 0.5–1 m. Panamanian specimens agree closely with previous descriptions of *Cerapusslayeri*, despite being juveniles. Notable exceptions include female uropod 1 peduncle with distoventral robust seta and pleopod 2 with plumose setae (simple in original description). We did not collect male specimens of *C.slayeri*, but diagnostic characters described previously include: antenna 1 peduncle 3 × as long as flagellum, peduncle articles 2 and 3 slender, subequal; flagellum 3- or 4-articulate. Antenna 2 flagellum tri-articulate. Pereonite 1 lateral keel absent. Gnathopod 2 carpus with process where the dactylus closes on propodus. Uropod 1 peduncle with large distoventral hook. Ethanol-preserved specimens retained purple coloration on most of the body, especially stripes on antennae.

**Figure 6.**
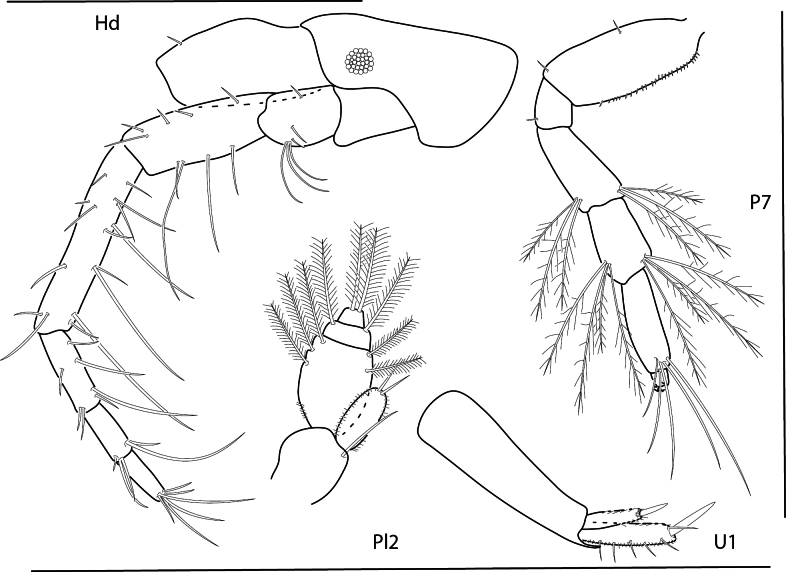
*Cerapusslayeri*, female, 2.0 mm, head, pleopod 2, pereopod 7, uropod 1. Scale bars: 0.5 mm.

##### ﻿ Cerapus thomasi

Taxon classificationAnimaliaAmphipodaIschyroceridae

Ortiz & Lemaitre, 1997

581F8F28-4CF2-5A8D-945B-146F11528DE8

Figs 7 , 31G

Cerapus sp.: Ortiz and Lemaitre 1994: 124.
Cerapus thomasi Ortiz & Lemaitre, 1997: 86–90, figs 15–20.

###### Material examined.

Panama • 1.6–2.7 mm • 1 ♀; Bocas del Toro, Crawl Caye; 9.2376°N, 82.1438°W, depth 1.5–3 m, among *Halimeda*, 11 Aug 2021; K.N. White leg.; USNM 1743949 • 1 ♀; Bocas del Drago; 9.4180°N, 82.3375°W; depth 2–3 m, among red algae, 9 Aug 2021; K.N. White leg.; USNM 1743950.

###### Diagnosis.

Head ocular lobe posterior margin even, reaching ~ 1/3 head length; rostrum slightly produced. Antenna 1 and 2 flagella bi- and tri-articulate. Pereopod 3 basis anteroproximal corner rectangular. Pleopod 2 outer ramus uni-articulate; inner ramus with two apical setae. Telson partially cleft. Ethanol-preserved specimens retained brown coloration on most of body, especially stripes on antennae.

**Figure 7.**
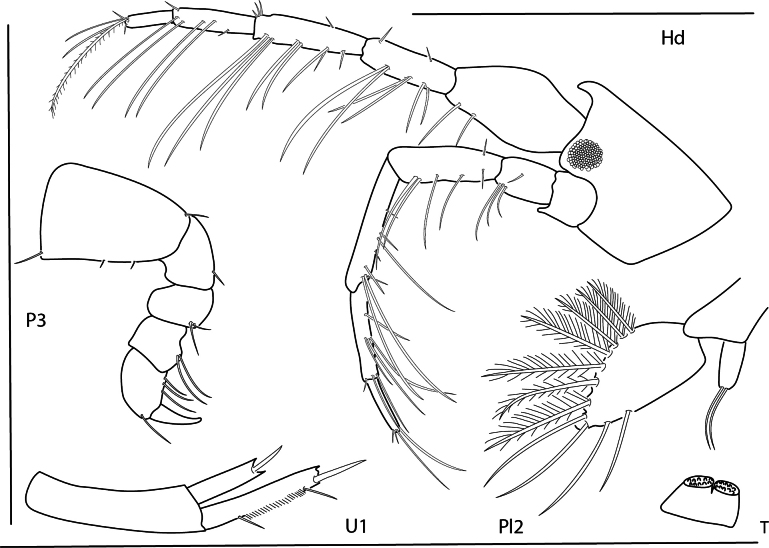
*Cerapusthomasi*, female, 1.6 mm, pereopod 3, head, uropod 1, pleopod 2, telson. Scale bars: 0.5 mm.

###### Distribution.

Colombia: Barú, Bahía de Cispatá, Gulf of Morrosquillo, South of Punta Comisario (Ortiz and Lemaitre 1994, 1997); Panama: Bocas del Toro (present study).

###### Ecology and remarks.

This species occurs among *Halimeda* and red algae at depths of 1.5–3 m. Panamanian specimens agree closely with previous descriptions of *Cerapusthomasi* with the following exceptions: antenna 2 number of flagellar articles and antennae color pattern.

##### ﻿Genus Ericthonius

Taxon classificationAnimaliaAmphipodaIschyroceridae

H. Milne Edwards, 1830

DD460909-C14E-56DA-BCF5-CFDA948C879B

###### Diagnosis.

Body subcylindrical. Antennae 1 and 2 peduncular articles 1–3 not broadly expanded, similar in width; antenna 1 accessory flagellum minute. Male coxa 2 distinctly separate from coxa 3; longer than wide in hyperadults. Gnathopod 1 subchelate, smaller than gnathopod 2. Male gnathopod 2 carpochelate. Pereopod 5 not geniculate. Pleopods 1–3 outer ramus thin; pleopods 2–3 rami not reduced, subequal. Uropod 2 bi-ramous. Uropod 3 uniramous, ramus with distal hook. Telson entire with dorsal recurved spines.

##### ﻿ Ericthonius brasiliensis

Taxon classificationAnimaliaAmphipodaIschyroceridae

(Dana, 1853)

FCE23884-1720-588C-B2D3-61E20D42BABC

Figs 8 , 31H

Pyctilus brasiliensis Dana, 1853: 976, fig. 5a–h.
Erichthonius brasiliensis : Bousfield 1973: 195, pl 59, fig. 2; Myers 1982: 200, 201, figs 136, 137; Myers and McGrath 1984: 382–385, figs 1, 2; Thomas 1993: 49, fig. 6; LeCroy 2007: 561, fig. 483.

###### Material examined.

Panama • 1.8–5.7 mm • 1 ♂; Bocas del Toro, Crawl Caye; 9.2504°N, 82.1316°W; depth 10 m, among coral rubble and red sponge; 7 Aug 2005; S. DeGrave, M. Salazar leg.; GCRL 6663 • 3 ♂, 2 ♀; Bocas del Toro, Hospital Point; 9.3048°N, 82.1316°W; depth 1.5 m, among sponges, coral rubble, and sand; 7 Aug 2005; T.A. Haney leg.; GCRL 6664 • 1 ♂, 5 ♀; Bocas del Toro, 100 m west of STRI dock; 14 m; 8 Aug 2005; T.A. Haney leg.; GCRL 6665 • 1 ♂, 1 ♀; Bocas del Toro, Isla San Cristobal; 9.2625°N, 82.1897°W; depth 0.2 m, 9 Aug 2005; S. LeCroy leg.; GCRL 6666 • 6 ♀; Bocas del Toro, Crawl Caye; 9.2376°N, 82.1438°W; depth 1.5–3 m, among *Halimeda*, 11 Aug 2021; K.N. White leg.; USNM 1743951 • 1 ♂, 1 ♀; Bocas del Toro, Crawl Caye; 9.2459°N, 82.1369°W; depth 1–4 m; 25 June 2023; K.N. White leg.; USNM 1743952 • 11 ♂, 17 ♀; Bocas del Toro, Hospital Point; 9.3333°N, 82.2185°W; depth 11 m, from buoy scrapings, 26 June 2023; K.N. White leg.; USNM 1743955 • 1 ♀; Bocas del Toro, Swan Cay; 9.4536°N, 82.3000°W; 27 June 2023; K.N. White leg.; USNM 1743956 • 3 ♀; Bocas del Toro, Cayo Zapatilla; 9.2699°N, 82.0587°W; depth 10–11 m; 28 June 2023; K.N. White leg.; USNM 1743953 • 4 ♂, 6 ♀; Cayo Zapatilla, Bocas del Toro; 9.2699°N, 82.0587°W; depth 0 m, in sand; 29 June 2023; K.N. White leg.; USNM 1743954 • 1 ♂; Crawl Caye, Bocas del Toro; 9.2502°N, 82.1318°W; depth 5–13 m, among coral rubble; 29 June 2023; K.N. White leg.; USNM 1743957.

###### Diagnosis.

Male gnathopod 1 basis widely expanded posterodistally. Coxa 2 anteroventral margin rounded, with vertical stridulating ridges, without long plumose setae. Male gnathopod 2 carpus anterodistal margin with two large distal projections. Coxa 3 evenly rounded distally, basis strongly expanded anterodistally. Pereopod 4 basis strongly expanded. Male pereopod 5 basis not produced into wing-like projection. Uropod 3 ramus slender.

###### Distribution.

Brazil: Rio De Janeiro (Dana 1853); U.S.A.: Cape Cod to Chesapeake Bay, Florida, Gulf states (Bousfield 1973); Italy: Thau, Napoli, Venezia, Lipari-Castello, Messina, Bosporus (Myers 1982); France: Banyuls-sur-Mer (Myers 1982); Cosmopolitan (Thomas 1993; LeCroy 2007); Panama: Bocas del Toro (present study).

###### Ecology and remarks.

This species occurs among coral rubble, red sponges, sand, and from buoy scrapings at depths of 0–11 m. Variation between Panamanian specimens has been seen in the following characters: size of posterior hump on basis; male gnathopod 2 basis posterior margin with fewer setae than previously described; uropod 3 more setose than previously described; color pattern (possibly due to differences in preservation). Variation within this species is further discussed in Myers and McGrath (1984). Panamanian specimens agree closely with specimens described by Bousfield (1973). Live specimens have brown stripes covering entire body with a red eye.

**Figure 8.**
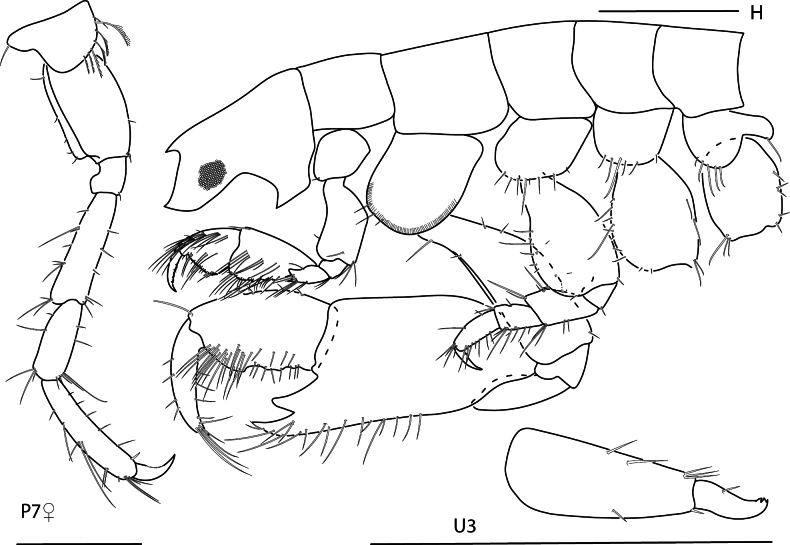
*Ericthoniusbrasiliensis*, female, 5.3 mm, pereopod 7; male 5.5 mm, habitus (in part); uropod 3. Scale bars: 0.5 mm.

#### ﻿Family Neomegamphopidae Myers, 1981

##### ﻿Genus Konatopus

Taxon classificationAnimaliaAmphipodaNeomegamphopidae

Barnard, 1970

0069DE27-78CD-55B3-B2C0-59755ACC8A85

###### Diagnosis.

Antenna 1 accessory flagellum short, bi-articulate. Eye slightly smaller than ocular lobe. Mandibular palp article 3 stout, clavate. Coxae overlapping. Male coxa 1 subovate, larger than remaining coxae. Female coxa 1 equally long as broad. Male gnathopod 1 carpus elongate with posterodistal tooth. Gnathopod 2 smaller than gnathopod 1, carpus longer than propodus. Uropod 1 peduncle with interramal spine. Uropod 3 biramous, rami slightly longer than peduncle, outer ramus with small barrel-shaped article. Telson broader than long, slightly concave.

##### ﻿ Konatopus tridens sp. nov.

Taxon classificationAnimaliaAmphipodaNeomegamphopidae

EDB299BE-18F1-5796-8439-238893029352

https://zoobank.org/EF59C1A6-B4A2-4E59-86C5-0C3E1DFC968A

**Figure 9.**
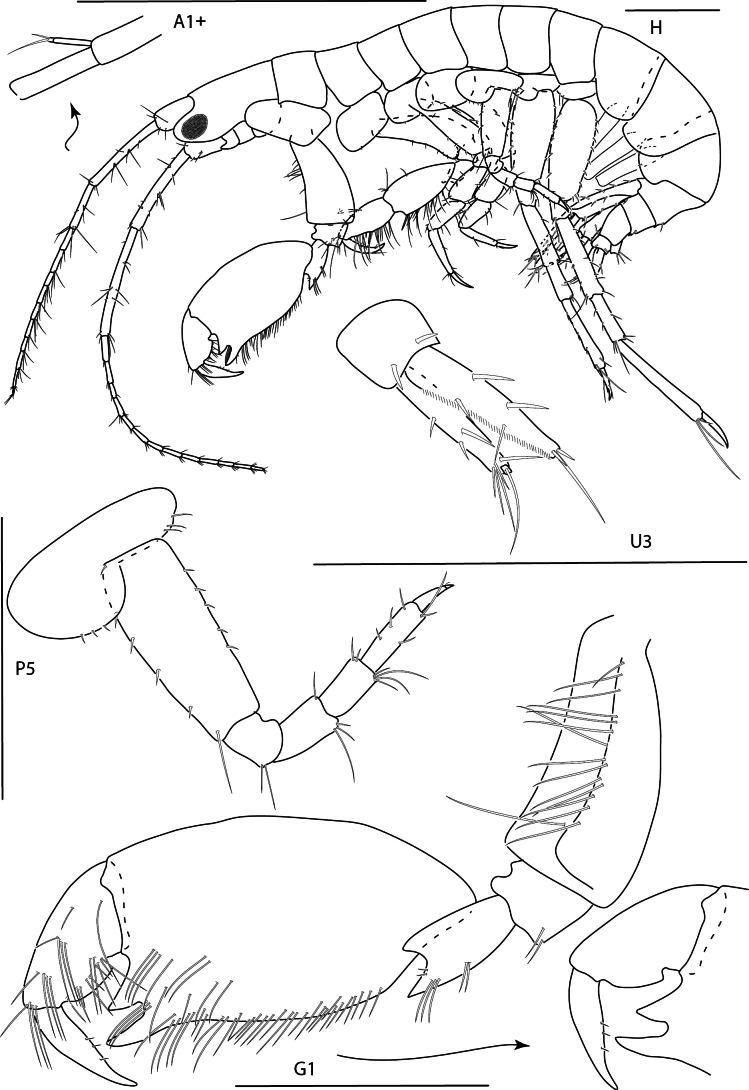
*Konatopustridens* sp. nov., male holotype, 4.2 mm, habitus, antenna 1 accessory flagellum, uropod 3, pereopod 5, gnathopod 1 medial. Scale bars: 0.5 mm.

**Figure 10.**
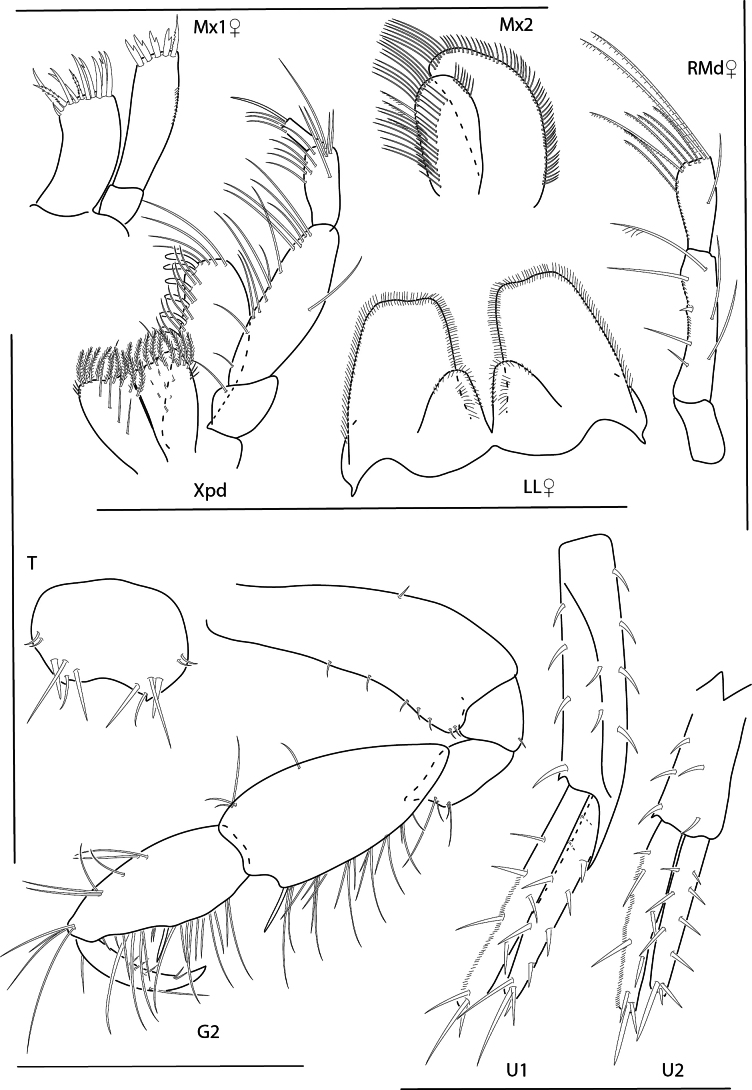
*Konatopustridens* sp. nov., female paratype, 4.7 mm, maxilla 1, lower lip, right mandibular palp; male holotype, 4.2 mm, maxilla 2, maxilliped, telson, gnathopod 2 lateral, uropod 1, uropod 2 (broken). Scale bars: 0.5 mm.

**Figure 11.**
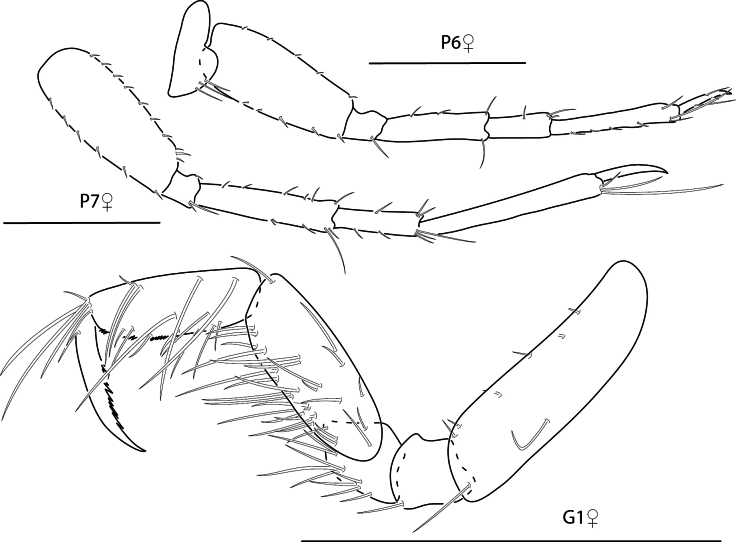
*Konatopustridens* sp. nov., female paratype, 4.7 mm, gnathopod 1 lateral, pereopod 6, pereopod 7. Scale bars: 0.5 mm.

Figs 9 , 10 , 11 , 32A

###### Type locality.

Bocas del Toro, Panama: Crawl Caye, 9.2459°N, 82.1369°W, depth 1–4 m, in sand.

###### Distribution.

Panama: Bocas del Toro (present study).

###### Material examined.

***Holotype***: Panama • 1 ♂, 4.2 mm; Bocas del Toro, Crawl Caye; 9.2459°N, 82.1369°W; depth 1–4 m, in sand; 25 June 2023; K.N. White leg.; USNM 1743958. ***Paratype***: Panama • 1 ♀, 4.7 mm; same station data as for preceding; USNM 1743959. ***Other material***: Panama • 1 ♂ juvenile, 3.0 mm; same station data as for preceding; USNM 1743960.

###### Diagnosis.

Male gnathopod 1 basis stout, merus with large anterodistal U-shaped excavation, carpus with three triangular anterodistal processes increasing in size distally, with deep U-shaped excavation between two lower processes, propodus subovate with large proximal notch. Pereopod 5 basis length 2.7 × width. Uropod 3 peduncle ~ 0.5 × length of outer ramus.

###### Description.

**Male** (holotype, 4.2 mm). ***Head.*** Ocular lobe rounded, eye ovate with many small ommatidia. Antenna 1 shorter than antenna 2, peduncle article 2 1.9 × length of article 1 and 3; flagellum setose with aesthetascs. Antenna 2 ~ 1.4 × length of antenna 1, flagellum moderately setose. Maxilliped inner plate with nine apical plumose setae, outer plate lined with long thin setae and six stout setae. Maxilla 1 missing. Maxilla 2 inner plate with row of facial setae, margin lined with dense setae. Upper lip missing. Lower lip rounded, apically setose. Mandibles similar; palp article 3 stout, clavate.

***Pereon.*** Coxae 1 large, subovate; coxae 2–4 subrectangular. Gnathopod 1 carpochelate; basis stout, with row of long facial setae; merus with large anterodistal U-shaped excavation, carpus with three triangular anterodistal processes increasing in size, with deep U-shaped excavation between two lower processes, propodus subovate with large proximal notch; dactylus thick, closing on carpus, marginally setose. Gnathopod 2 subchelate, much smaller than gnathopod 1; basis widened distally, anterior margin with sparse setae, carpus distally setose with one robust anterodistal seta, propodus distally setose with one distal robust seta. Pereopods 3–7 basis and propodus narrow, elongate. Pereopods 3 and 4 dactylus narrow, elongate. Pereopod 5 dactylus short, stout. Pereopods 6 and 7 much longer than pereopod 5; dactylus long, narrow.

***Pleon.*** Epimera 1–3 rounded, with few distal setae. Uropod 1 with interramal spine, peduncle subequal in length with inner ramus, both margins lined with robust setae, with facial row of robust setae; inner ramus 1.1 × length of outer ramus, lined with robust setae, medial margin lined with fine setae, apical margin with three robust setae; outer ramus both margins lined with robust setae, apical margin with four robust setae. Uropod 2 peduncle broken, with several robust setae, medio-distal margin with acute point; inner ramus 1.2 × length of outer ramus, both margins lined with robust setae, medial margin lined with fine setae, apical margin with three robust setae; outer ramus both margins lined with robust setae, apical margin with three. Uropod 3 peduncle 0.5 × length of inner ramus, with two distal robust setae, medio-distal margin with acute point; inner ramus 1.1 × length of outer ramus, both margins with few robust setae, lateral margin lined with fine setae, apical margin with three robust setae; outer ramus bi-articulate, article 1 margins with few robust setae, apical margin with four robust setae, article 2 with one long seta. Telson apical margin slightly concave with acute lateral points, each point surrounded by four robust setae, lateral margins each with two setae.

**Female** (paratype, 4.7 mm). Similar in all aspects to the male with the exception of the following: Lower lip outer lobes lined with setae; mandibular lobes long, pointed. Maxilla 1 inner plate apical margin lined with bifurcate setae; palp bi-articulate, article 2 apical margin with four bifurcate robust setae and one serrate robust seta, outer margin with fine setules. Gnathopod 1 weakly subchelate, merus, carpus, propodus, and dactylus densely setose, propodus and dactylus lateral margins lined with fine setae. Gnathopod 2 missing.

###### Etymology.

After the Latin *tridens*, meaning fork with three tines and referring to the three triangular process on the gnathopod 2 carpus of males of this species.

###### Ecology and remarks.

This species occurs among sand at depths of 1–4 m. This species most closely resembles *Konatopustulearensis* Ledoyer, 1982 in sharing the male gnathopod 1 stout basis with a subovate propodus with proximal notch. The new species can be distinguished from *K.tulearensis* based on male gnathopod 1 merus with short acute distal lobe (vs large lobe), carpus with three triangular anterodistal processes increasing in size, lowest process with deep U-shaped excavation (vs 1 process and small excavation), propodus subovate with large proximal notch (vs subrectangular with small proximal notch), and pereopod 5 basis length 2.7 × width (vs 1.3 × width). The new species can be distinguished from all other described *Konatopus* species based on the shape of the gnathopod 1 carpus: with one large distal subacute process in *Konatopuslatipalmus* Ledoyer, 1979; with one slight distal process in *Konatopuspaao* J.L. Barnard, 1970; and produced into a rounded lobe distally in *Konatopusstoreyae* Myers, 2002. Live specimens are white in color with brown splotches.

##### ﻿Genus Varohios

Taxon classificationAnimaliaAmphipodaNeomegamphopidae

J.L. Barnard, 1979

B5D11B6C-3F69-5B0D-AB25-77482BB7ECA6

###### Diagnosis.

Male coxa 1 subquadrate. Male gnathopod 1 chelate, carpus and propodus fused, dactylus with posteroproximal tooth. Gnathopod 2 smaller than gnathopod 1, propodus longer than carpus. Uropod 1 with large interramal spine. Uropod 3 rami subequal in length with peduncle, outer ramus with small barrel-shaped article 2.

##### ﻿ Varohios topianus

Taxon classificationAnimaliaAmphipodaNeomegamphopidae

Barnard, 1979

79945FC7-CA22-5A34-9F82-4F14898DE792

Figs 12 , 32B

Varohios topianus Barnard, 1979: 35–37, figs 13, 14.

###### Material examined.

Panama • 2.3 mm • 1 ♂; Bocas del Toro, Crawl Caye; 9.2699°N, 82.0587°W, depth 1–4 m, among coral rubble; K.N. White leg.; USNM 1743961.

###### Diagnosis.

Gnathopod 1 basis with rows of anterior, posterior, and facial setae; dactylus posteroproximal tooth longer than wide. Male pereopod 5 basis length 1.4 × width. Uropod 1 interramal spine subequal in length with peduncle; inner ramus with robust setae on both margins, lateral margin lined with small setae; outer ramus with two robust setae on lateral margin. Uropod 3 rami subequal, rami subequal to peduncle, outer ramus with small barrel shaped article 2. Telson apex convex, dorsal surface with excavation. Live specimens are white in color with brown splotches.

**Figure 12.**
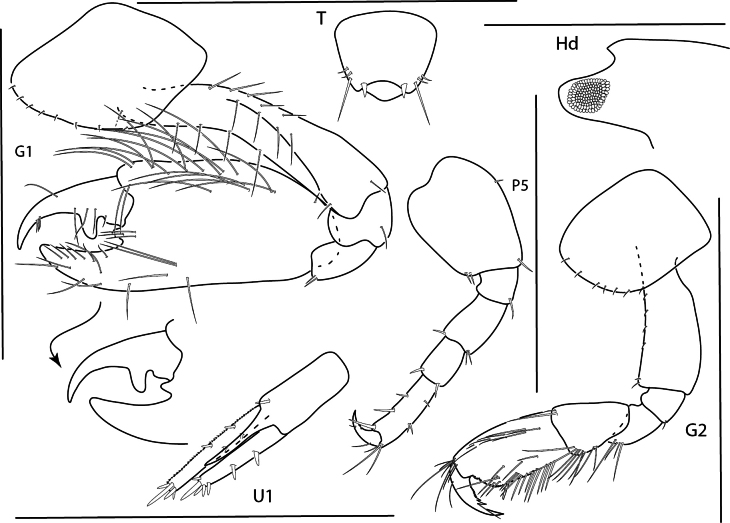
*Varohiostopianus*, male, 2.3 mm, gnathopod 1 medial, telson, uropod 1, pereopod 5, head, gnathopod 2 medial. Scale bars: 0.5 mm.

###### Distribution.

U.S.A.: Gulf of California (Barnard 1979); Ecuador: Galápagos Islands (Barnard 1979); Panama: Bocas del Toro (present study).

###### Ecology and remarks.

This species occurs among coral rubble at depths of 1–4 m. Panamanian specimen agrees closely with specimens described by Barnard (1979) with the exception of fewer setae on the gnathopod 1 basis and carpus/propodus, fewer robust setae on uropod 1 rami; both of which could be variation based on size. This species has previously only been collected from the Pacific Ocean.

#### ﻿Family Photidae Boeck, 1871

##### ﻿Genus Audulla

Taxon classificationAnimaliaAmphipodaPhotidae

Chevreux, 1901

E8F5C058-D0F0-5BFC-A01C-14D2A958B1D0

###### Diagnosis.

Head ocular lobe narrowly rounded anteriorly, inferior antennal sinus deeply recessed for insertion of antenna 2. Antenna 1 peduncle article 3 subequal to article 1 in length, accessory flagellum 5- or 6-articulate. Male antenna 2 flagellum dorsoventrally flattened. Male gnathopod 2 minutely chelate, propodus subrectangular, palm uncurving, with distal margin extending anteriorly. Female gnathopod 2 larger than gnathopod 1; propodus anterior margin with dense rows of setae. Uropod 3 biramous, rami subequal in length, peduncle longer than telson.

##### ﻿ Audulla chelifera

Taxon classificationAnimaliaAmphipodaPhotidae

(Chevreux, 1901)

47557D04-FEB1-58BC-8DDA-6A9ED833BE17

Figs 13 , 33C

Gammaropsis chelifera Chevreux, 1901: 432–436, figs 56–65.
Eurystheus lina : Kunkel 1910: 81–83, fig. 31.
Eurystheus semichelatus : K.H. Barnard 1957: 8, fig. 5.
Gammaropsis lina : Lazo-Wasem and Gable 1987: 331–335, figs 7–9.
Audulla chelifera : Thomas and Barnard 1987: 364–369, figs 1–4; LeCroy 2000: 125, fig. 163.

###### Material examined.

Panama • 4.2–5. 8 mm • 7 ♂, 5 ♀ • Bocas del Toro, Lime Point; 9.414°N, 82.3323°W; depth 0.2–0.5 m, among coral rubble and red algae; 5 Aug 2005; S. DeGrave, M. Salazar leg.; GCRL 6667.

###### Diagnosis.

Male antenna 2 flagellum dorsoventrally flattened. Male gnathopod 2 minutely chelate, basis anterior and posterior margins with long setae; propodus subrectangular with rounded projection at palmar angle, densely setose. Female gnathopod 1 smaller than gnathopod 2; ischium, merus, carpus, with plumose setae. Gnathopod 2 propodus anterior margin densely setose. Uropod 3 rami subequal in length.

###### Distribution.

South Africa: St. Helena Bay (K.H. Barnard 1957); Bermuda: exact location unknown (Kunkel 1910, Lazo-Wasem and Gable 1987); USA: Florida (LeCroy 2000), Gulf of Mexico (LeCroy et al. 2009); Caribbean Sea (Barnard and Karaman 1991); Belize: Curlew Cay (Thomas and Barnard 1987); Mexico: Yucatan (McKinney 1977); Seychelles Islands: La Digue (Chevreux 1901; Ledoyer 1982; Barnard and Karaman 1991); Panama: Bocas del Toro (present study).

**Figure 13.**
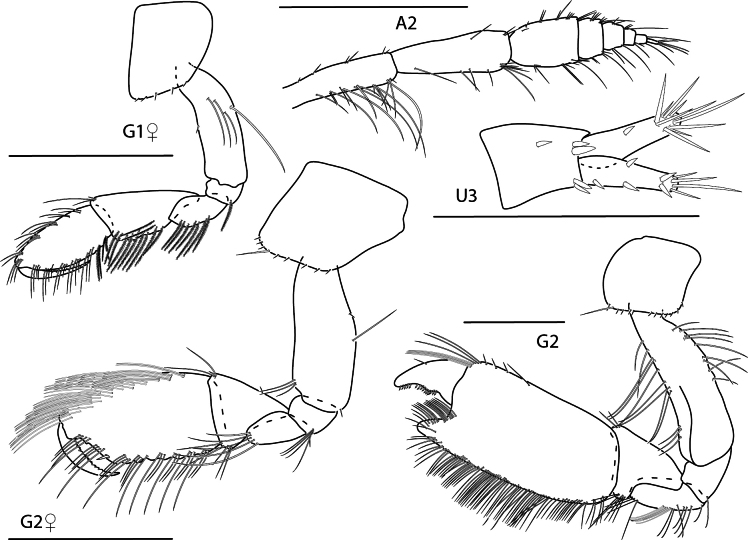
*Audullachelifera*, female, 4.8 mm, gnathopod 1 medial, gnathopod 2 medial; male, 4.9 mm, antenna 2, uropod 3, gnathopod 2lateral. Scale bars: 0.5 mm.

###### Ecology and remarks.

This species occurs among coral rubble and red algae at depths of 0.2–5.8 m. Panamanian specimens agree closely with previous descriptions of *Audullachelifera*.

##### ﻿Genus Latigammaropsis

Taxon classificationAnimaliaAmphipodaPhotidae

Myers, 2009

56B09CAB-1E11-53D2-A24E-3DD88B83BC1B

###### Diagnosis.

Head cephalic lobes rounded, anteroventral margin surpassing posterior margin of eye; eyes at least partially situated within cephalic lobe. Antenna 2 flagellum longer than peduncle article 5. Uropod 3 peduncle stout; outer ramus bi-articulate, second article vestigial, subtruncate with two fine setae; inner ramus subequal to or shorter than outer ramus, narrowing distally, with one stout apical seta.

##### ﻿ Latigammaropsis atlantica

Taxon classificationAnimaliaAmphipodaPhotidae

(Stebbing, 1888)

6DE73836-96C2-5155-8E3E-158B212081CB

Figs 14 , 32D

**Figure 14.**
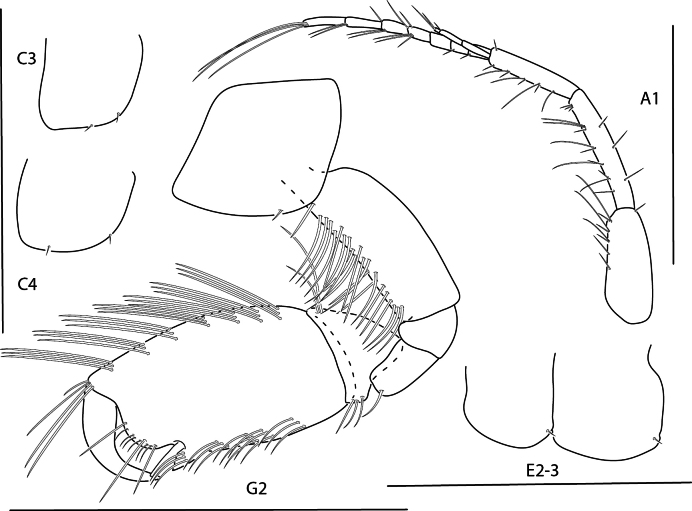
*Latigammaropsisatlantica*, male, 4.9 mm, coxa 3, coxa 4, gnathopod 2 medial, epimera 2 and 3; male, 2.3 mm, antenna 1. Scale bars: 0.5 mm.

Gammaropsis atlantica Stebbing, 1888: 1101, fig. 114; Myers 1985: 80, fig. 60; LeCroy 2000: 135, fig. 176.
Gammaropsis zeylanicus : Walker 1904: 282, 283, fig. 41.
Gammaropsis gardineri : Walker 1905: 929, 930, figs 11–14, 16–17.
Eurystheus atlantica : Stebbing 1906: 611.
Latigammaropsis atlantica : Myers 2009: 777.

###### Material examined.

Panama • 2.4–3 mm • 3 ♂; Bocas del Toro, Hospital Point, Cayo Solarte; 9.3336°N, 82.2188°W; depth 15 m, among coral rubble and *Halimeda*; 6 Aug 2005; S. DeGrave leg.; GCRL 6668 • 2 ♂; Bocas del Toro, Crawl Caye, 9.2459°N, 82.1369°W; depth 1–4 m, among coral rubble; 25 Jun 2023; K.N. White leg.; USNM 1743962.

###### Diagnosis.

Male gnathopod 2 propodus distinctly longer than carpus, robust seta present at palmar angle; dactylus subequal in length with propodus palm. Coxae 3 and 4 subquadrate. Epimera 2 and 3 posteroventral margins subquadrate with weak notches.

###### Distribution.

Cape Verde Islands: Saint Vincent (Stebbing 1888); Sri Lanka (Walker 1904); Maldives: Hulule, Fadifolu, Mahlosmadulu Atoll, Minikoi (Walker 1905); Cape Verde Islands: St. Vincent (Stebbing 1906); Fiji: Momi Bay, Mburelevu, Nananui Ra (Myers 1985); USA: Florida (LeCroy 2000); Panama: Bocas del Toro (present study).

###### Ecology and remarks.

This species occurs among coral rubble and *Halimeda* at depths of 1–15 m. Panamanian specimens agree closely with LeCroy (2000) with the exception of antenna 1, accessory flagellum tri-articulate (vs 5- or 6-articulate). The smaller size of our specimen suggests this character is variable based on size. No females were collected in this study, but LeCroy (2000) reported a convex gnathopod 2 palmar margin of the propodus in females. Ethanol-preserved specimens retained brown coloration on head and pereon. There are many reports of this species worldwide, but they most likely represent a species-group and material from around the world needs to be examined.

##### ﻿Genus Photis

Taxon classificationAnimaliaAmphipodaPhotidae

Krøyer, 1842

F6B71422-21DB-51BF-A7E4-483179146633

###### Diagnosis.

Antenna 1 accessory flagellum vestigial or absent. Coxae 1 and 2 subequal in length with coxae 3 and 4. Gnathopod 2 sexually dimorphic. Male gnathopod 2 subchelate; dactylus slender. Female gnathopod 2 propodus anterior margin sparsely to moderately setose. Female pereopods 3 and 4 oostegites broadly expanded, longer than basis. Urosomites separate. Uropods 1 and 2 inner ramus lanceolate with apical robust setae. Uropod 3 inner ramus minute.

##### ﻿ Photis butalus sp. nov.

Taxon classificationAnimaliaAmphipodaPhotidae

8805722B-ADB0-5982-B4D0-A61C10B39DCA

https://zoobank.org/2C9CC06A-324D-4E52-A321-E016006F291E

**Figure 15.**
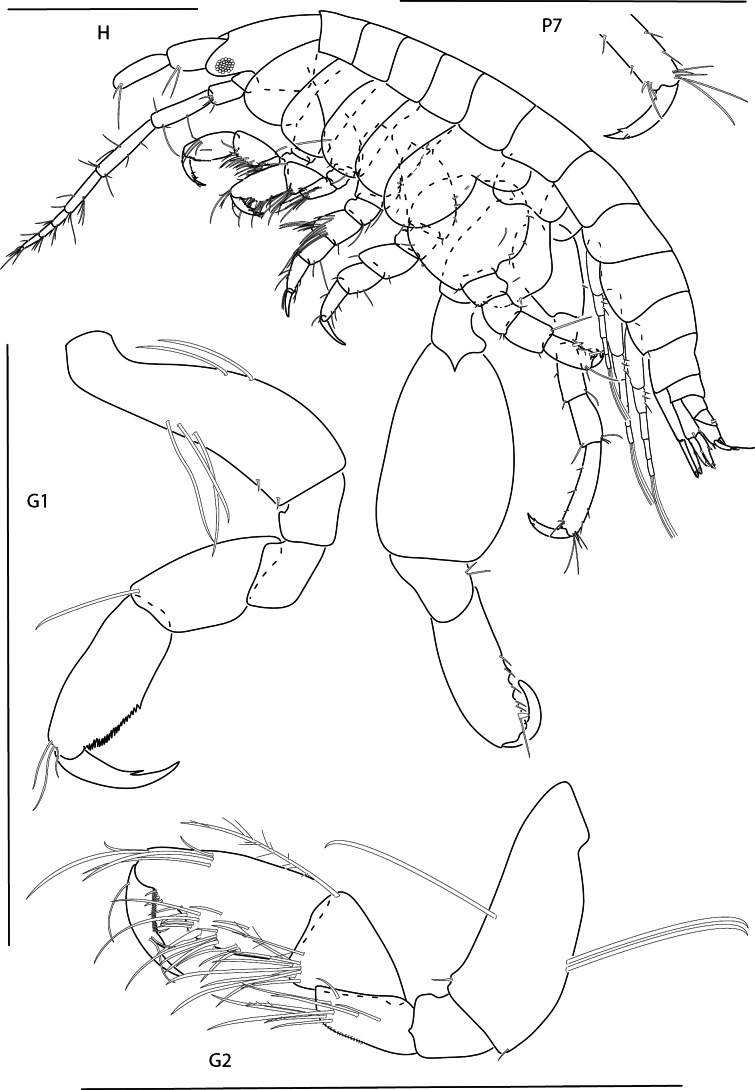
*Photisbutalus* sp. nov., male holotype, 2.1 mm, habitus, pereopod 7 dactylus, gnathopod 1 medial, gnathopod 2 medial. Scale bars: 0.5 mm.

**Figure 16.**
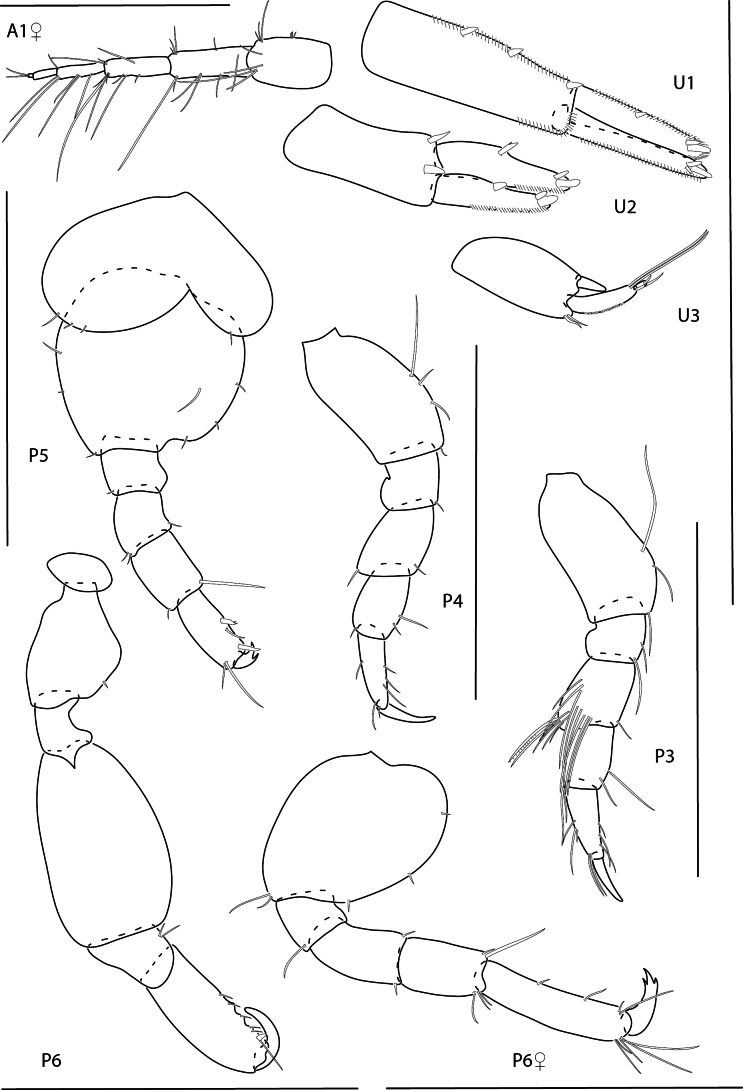
*Photisbutalus* sp. nov., female paratype, 2.3 mm, antenna 1, pereopod 6; male holotype, 2.1 mm, uropod 1, uropod 2, uropod 3, pereopod 5, pereopod 4, pereopod 3, pereopod 6. Scale bars: 0.5 mm.

**Figure 17.**
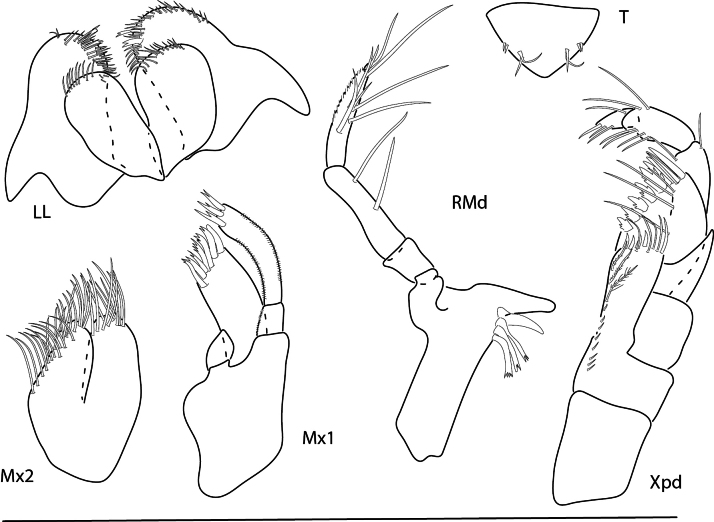
*Photisbutalus* sp. nov., male holotype, 2.1 mm, lower lip, maxilla 2, maxilla 1, right mandible, telson, maxilliped. Scale bars: 0.5 mm.

Figs 15 , 16 , 17 , 32E

###### Type locality.

Bocas del Toro, Panama: Swan Cay; 9.4533°N, 82.2983°W, depth 3 m, among brown algae, hydroids, and filamentous algae.

###### Material examined.

***Holotype***: Panama • 1 ♂, 2.1 mm; Bocas del Toro, Swan Cay; 9.4533°N, 82.2983°W; depth 3 m, among brown algae, hydroids, and filamentous algae; 4 Aug 2005; T.A. Haney leg.; USNM 1743981. ***Paratype***: Panama • 1 ♀, 2.3 mm; same station data as for preceding; GCRL 6669. ***Other material***: Panama • 1 ♂,1.8 mm; 4 ♀, 2–2.6 mm; 1 juvenile, mm; same station data as for preceding; GCRL 6670.

###### Diagnosis.

Eye well developed, not touching outer margin of ocular lobe. Gnathopod 1 propodus palm entire. Pereopod 6 of adult male greatly enlarged; merus ovate; propodus thick, width 0.4 × length, palmar margin minutely serrate with short setae, one short subtriangular seta present; dactylus apical margin subacute. Pereopod 7 dactylus with posterior and anterior accessory claws.

###### Description.

**Male** (holotype, 2.1 mm). ***Head*.** Eye well developed, not touching outer margin of ocular lobe. Antenna 2 flagellum 5-articulate. Maxilliped inner plate lined with setae along inner margin, two rows of apical setae present, outer plate with row of four thick setae, and six sagittate-shaped setae. Lower lip inner lobes rounded, outer lobes with large gape, apically setose, with few thick bifurcate setae; inner plate length 0.8 × length of outer plate, apically setose. Maxilla 1 inner plate small, bare; outer plate with two rows of five apical bifurcated robust setae; palp bi-articulate, article 2 lined with marginal setules, apical margin with three bifurcated robust setae. Maxilla 2 inner lobe with two rows of marginal setae; outer lobe with two rows of apical setae. Mandibles similar, molar small; palp tri-articulate, article 3 lined with fine setules.

***Pereon*.** Coxae sparsely setose. Coxa 1–4 subrectangular, longer than wide. Gnathopod 1 subchelate; basis anterior and posterior margins with few long setae; merus with posterodistal cluster of setae; carpus subequal in length to propodus, densely setose; propodus palm convex, serrate, proximal margin with one large robust seta; dactylus lined with minute stout setae, with one serration at distal end. Gnathopod 2 subchelate; basis anterior and posterior margins each with one long seta and one short distal seta; merus with posterodistal bunch of long setae, posterodistal margin with fine setules; carpus subtriangular, with posterodistal cluster of fine setae; propodus palm with weak excavation, palmar robust seta present; dactylus extending past excavation of propodus palm, lined with minute stout setae, with one distal serration. Pereopods 3 and 4 bases thick, posterodistal margin with few long setae; dactylus apically subacute. Pereopod 3 merus anterodistal margin densely setose. Pereopod 5 basis nearly circular, sparsely setose; propodus with two posterodistal robust setae; dactylus stout with accessory claw. Pereopod 6 of adult male greatly enlarged; merus ovate; propodus thick, width 0.4 × length, palmar margin minutely serrate with short setae, one short subtriangular robust seta present; dactylus apical margin subacute. Pereopod 7 basis narrowing distally; remaining articles slender; propodus distal margin sparsely lined with short setae and one short subtriangular robust seta; dactylus with posterior and anterior accessory claws.

***Pleon*.** Uropod 1 peduncle 1.3 × length of inner ramus, with three marginal robust setae and lined with fine setules; inner ramus 1.1 × length of outer ramus, with one marginal stout seta, lined with marginal setules, apex with two robust setae; lined with marginal setules, apex with three robust setae. Uropod 2 peduncle with two distal robust setae, subequal in length with inner ramus; inner ramus 1.2 × length of outer ramus, one marginal robust setae, inner margin lined with setules, apex with two robust setae; outer ramus outer margin lined with setules, with one marginal robust setae, apex with two robust setae. Uropod 3 peduncle 3.9 × length of inner ramus, with two distal setae; inner ramus 0.4–0.5 × length of outer ramus, bare; outer ramus bi-articulate, first article outer margin lined with setules, with three long distal setae; second article with one distal seta. Telson narrowing distally, apically rounded with four dorsal setae.

**Female** (paratype, 2.3 mm). Similar in all aspects to the male with the exception of the following: pereopod 6 merus not enlarged, propodus width 0.2 × length, smooth; dactylus apically acute with accessory claws.

###### Etymology.

After the Latin *bu*, meaning large and *talus*, meaning ankle, heel, die and referring to the greatly enlarged pereopod 6, specifically the thickened propodus, of males of this species.

###### Distribution.

Panama: Bocas del Toro (present study).

###### Ecology and remarks.

This species occurs among brown algae, hydroids, and filamentous algae at a depth of three meters.

*Photisbutalus* sp. nov. is similar to *Photistrapherus* Thomas & Barnard, 1991 and *Photiselephantis* Barnard, 1962 based on the enlarged male pereopod 6. The new species differs from *P.trapherus* in the following characters: eye not touching outer margin of ocular lobe (vs touching); entire gnathopod 1 propodus palm (vs slightly excavate); male pereopod 6 merus ovate (vs subrectangular), and propodus thick, posterior margin serrate (vs thin, smooth). The new species differs from *P.elephantis* in the following characters: ocular lobe pronounced, rounded (vs small, subquadrate); male pereopod 6 basis posteriorly rounded (vs posterior margin with acute distal point), merus posteriorly rounded (vs posterodistal margin produced into lobe), and propodus posterior margin serrate (vs smooth). This species is also similar to *Photis* sp. E LeCroy, 2000 but differs in the following characters: eye well developed (vs poorly developed); male pereopod 6 greatly enlarged (vs slightly enlarged), merus ovate (vs subrectangular with large posterodistal projection). This species is easily distinguishable from all remaining described *Photis* species based on the enlarged male pereopod 6. Ethanol-preserved specimens retained brown specks of color.

##### ﻿ Photis bulla sp. nov.

Taxon classificationAnimaliaAmphipodaPhotidae

40FE8260-6BFB-5D29-856D-65C5B21A136B

https://zoobank.org/E0C60C56-2A5F-402C-B5C5-07CA6F32BF35

**Figure 18.**
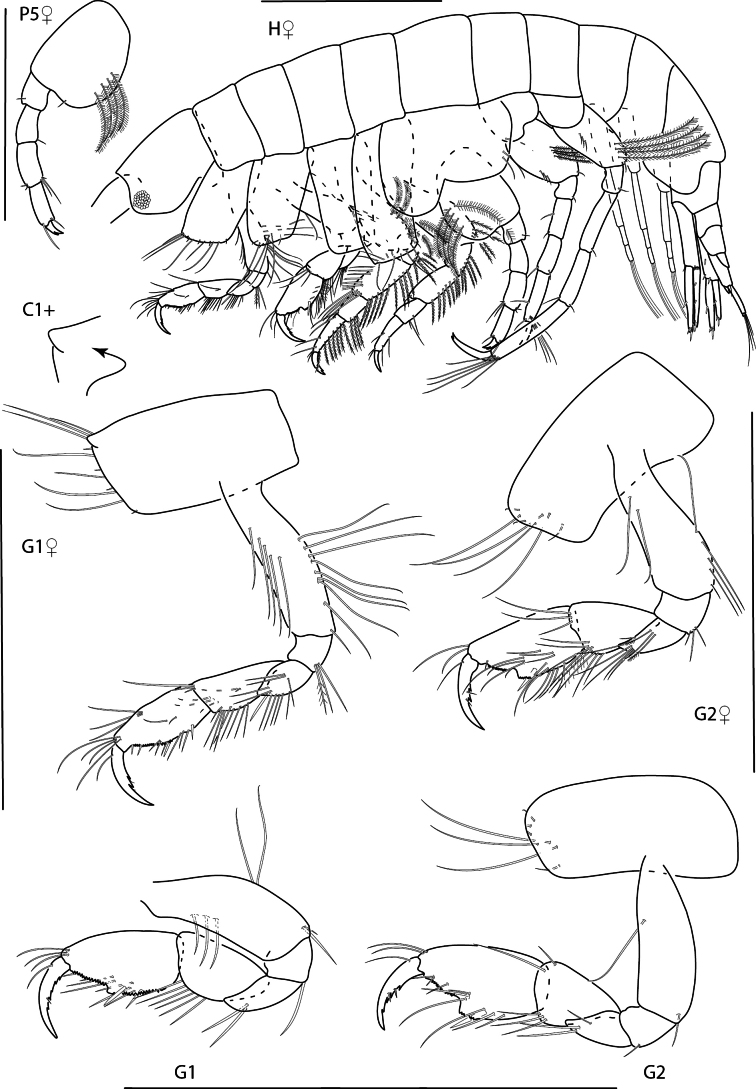
*Photisbulla* sp. nov., female holotype, 2.4 mm, pereopod 5, habitus, gnathopod 1 medial, gnathopod 2 medial; male paratype, 1.2 mm, gnathopod 1 medial, gnathopod 2 medial. Scale bars: 0.5 mm.

**Figure 19.**
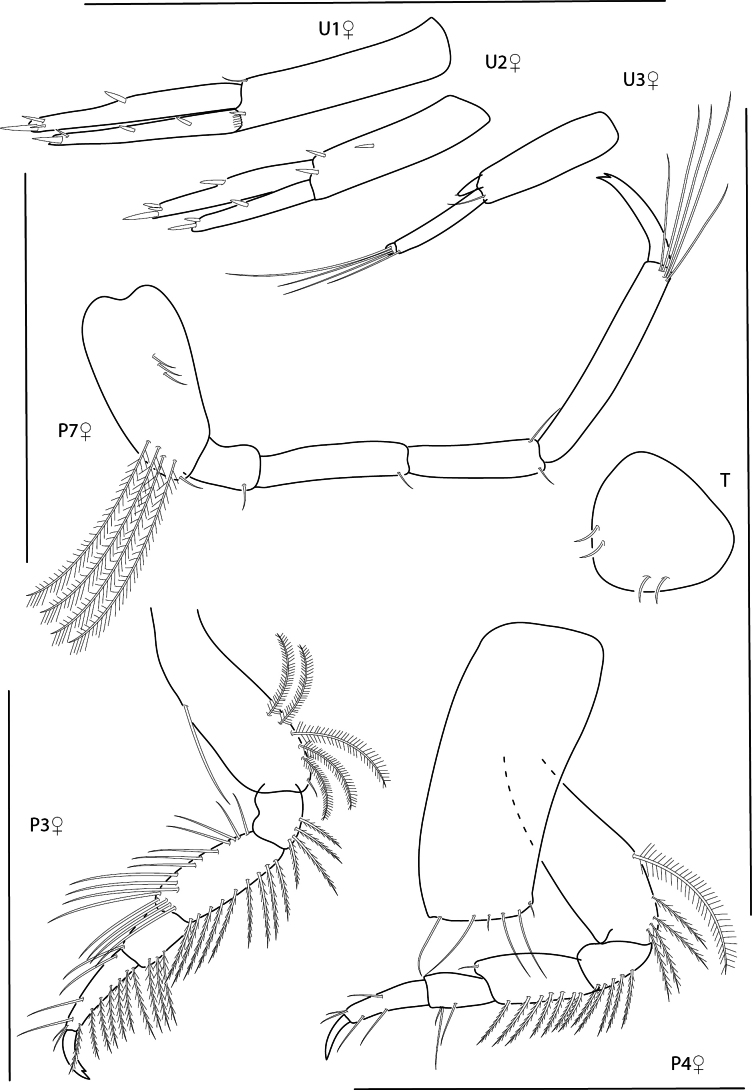
*Photisbulla* sp. nov., female holotype, 2.4 mm, uropod 1, uropod 2, uropod 3, pereopod 7, pereopod 3, pereopod 4; male paratype, 1.2 mm, telson. Scale bars: 0.5 mm.

**Figure 20.**
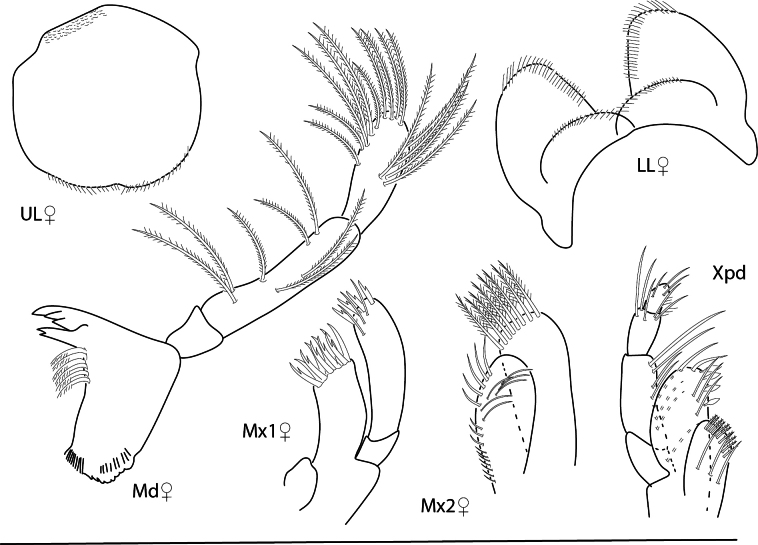
*Photisbulla* sp. nov., female holotype, 2.4 mm, upper lip, lower lip, mandible, maxilla 1, maxilla 2; male paratype, 1.2 mm, maxilliped. Scale bars: 0.5 mm.

Figs 18 , 19 , 20 , 32F

Photis sp. C: LeCroy 2000: 157, fig. 185.

###### Type locality.

Bocas del Toro, Panama: Crawl Caye, 9.2475°N, 82.1290°W, depth 1.5–3 m, among *Halimeda*.

###### Material examined.

***Holotype***: Panama • 1 ♀, 2.4 mm; Bocas del Toro, Crawl Caye; 9.2475°N, 82.1290°W; depth 1.5–3 m, among coral rubble; 11 Aug 2021; K.N. White leg.; USNM 1743963. ***Paratype***: Panama • 1 ♂, 1.2 mm; Bocas del Toro, Crawl Caye; 9.2376°N, 82.1438°W; depth 5 m, among *Halimeda*; 12 Aug 2021; K.N. White leg; USNM 1743964. ***Other material***: Panama • 2 ♀, 1.8–2.2 mm; Bocas del Toro, Crawl Caye; 9.2376°N, 82.1438°W; depth 4.6 m, among coral rubble; 11 Aug 2021; K.N. White leg.; USNM 1743966 • 1 juvenile, 1.2 mm; Bocas del Toro, Crawl Caye; 9.2475°N, 82.1290°W; depth 5 m, among *Halimeda*; 12 Aug 2021; K.N. White leg; USNM 1743965.

###### Diagnosis.

Head ocular lobe rounded distally. Coxae 1–4 ventral margins lined with long setae; coxa 1 anteroventral margin slightly produced with gap in marginal setae. Gnathopod 1 carpus slightly shorter than propodus in length, anterior margin subquadrate proximally. Gnathopod 2 propodus with stout robust seta at palmar angle, dactyl, flexor margin serrate. Pereopods 5–7 basis anterior margin with row of long submarginal plumose setae. Pereopods 6 and 7 propodus each with posterodistal cluster of setae surpassing length of dactylus. Uropod 3 inner ramus with minute spinule.

###### Description.

(Female 2.4 mm). ***Head*.** Head, ocular lobe rounded distally. Maxilliped, inner plate apical margin lined with plumose robust setae; outer plate with row of marginal sagittate-shaped robust setae and row of submarginal simple setae; palp 4-articulate, articles 1–3 inner margins lined long thin setae, article 4 with stout setae. Upper lip apically setose. Lower lip inner and outer lobes rounded, apically setose; outer lobe with few thick bifurcate setae. Maxilla 1 outer plate with four apical robust setae and four slender setae; inner plate with nine apical robust setae. Maxilla 2 inner plate outer margin lined with setae, submarginal row of long setae; outer plate apical margin lined with long plumose setae. Mandibles similar, molar small; palp tri-articulate, articles 2 and 3 with plumose setae.

***Pereon*.** Coxae 1–4 lined with long ventral setae; coxa 1 anteroventral margin slightly produced with gap in marginal setae. Gnathopod 1 subchelate; basis unexpanded, margins lined with long setae; carpus slightly shorter than propodus; propodus palm serrate with one robust seta at proximal margin; dactylus serrate. Gnathopod 2 similar to gnathopod 1 with deep palmer excavation just before robust seta at palmar angle. Pereopods 3 and 4 posterior margins of articles 2–4 lined with long plumose setae. Pereopod 5 basis length 1.2 × width; dactylus stout. Pereopods 5–7 basis with row of submarginal plumose setae. Pereopods 6 and 7 propodus each with posterodistal cluster of setae surpassing length of dactylus; dactylus with accessory claw.

***Pleon*.** Epimera 1–3 distal margins rounded. Uropod 1 peduncle subequal in length with inner ramus; inner ramus 1.1 × length of outer ramus, both rami with marginal and apical robust setae. Uropod 2 peduncle 1.1 × length to inner ramus, peduncle with two strong distomedial robust setae; inner ramus 1.3 × length of outer ramus, each ramus with one marginal robust seta and two or three apical robust setae. Uropod 3 peduncle 5.3 × length of inner ramus; inner ramus 0.3 × length of outer ramus, with small apical spinule; outer ramus bi-articulate, terminal article with many long setae. Telson apex rounded, subtriangular.

**Male** (paratype 1.2 mm). Similar in all aspects to the female with the exception of the following: Gnathopod 1 propodus palm with excavation. Gnathopod 2 with process near insertion of dactylus. Pereopods 3, 5, and 7 missing. Pereopod 4 sparsely setose. Pereopod 6 lacking plumose setae on basis.

###### Etymology.

After the Latin *bulla*, meaning knob, boss, stud, bubble and referring to the anteroventral projection on the anteroventral margin of coxa 1 of this species.

###### Distribution.

U.S.A.: Florida: Hutchinson Island to the Florida Keys, Florida Bay to Perdido Key (LeCroy 2000); Panama: Bocas del Toro (present study).

###### Ecology and remarks.

This species occurs among coral rubble and *Halimeda* at depths of 1.5–3 m. Panamanian specimens agree closely with the description of *Photis* sp. C of LeCroy (2000) with the exception of unequal rami on uropod 1 and a more rounded telson apex. Panamanian specimens have a small spinule on the inner ramus of uropod 3, which LeCroy noted as variable. *Photisbulla* sp. nov. most closely resembles *Photisspinicarpa* Shoemaker, 1942 based on gnathopod 1 carpus subquadrate proximally, gnathopod 2 with stout seta at palmar angle, gnathopod 2 dactyl flexor margin serrate, and pereopod 5 basis anterior margin with plumose setae but can be distinguished based on the absence of robust setae on upper proximal margin of gnathopod 1 carpus, male gnathopod 2 basis and carpus without anterodistal process, and pereopod 3 merus unexpanded. *Photisbulla sp. nov.* differs from *Photisprobolion* sp. nov. in the following characters: coxa 1 with setae; male coxae 3 and 4 without stridulating ridges; female pereopod 5 with submarginal plumose setae; uropods 1 and 2 outer ramus with marginal setae. *Photisbulla* sp. nov. differs from all remaining described *Photis* species in having coxa 1 anteroventral margin slightly produced with gap in marginal setae. Additionally, the new species differs from *Photismelanica* and *Photisbutalus* sp. nov. in having a posterodistal cluster of setae surpassing length of dactylus on pereopods 6 and 7. Live specimens are a mottled purple-brown color with purple-brown stripes on distal ends of antennae.

##### ﻿ Photis probolion sp. nov.

Taxon classificationAnimaliaAmphipodaPhotidae

3B09027D-02D2-55A7-8E8C-7646A224CF88

https://zoobank.org/7428F59B-F31A-49AA-B069-60CEC6B3C3AF

**Figure 21.**
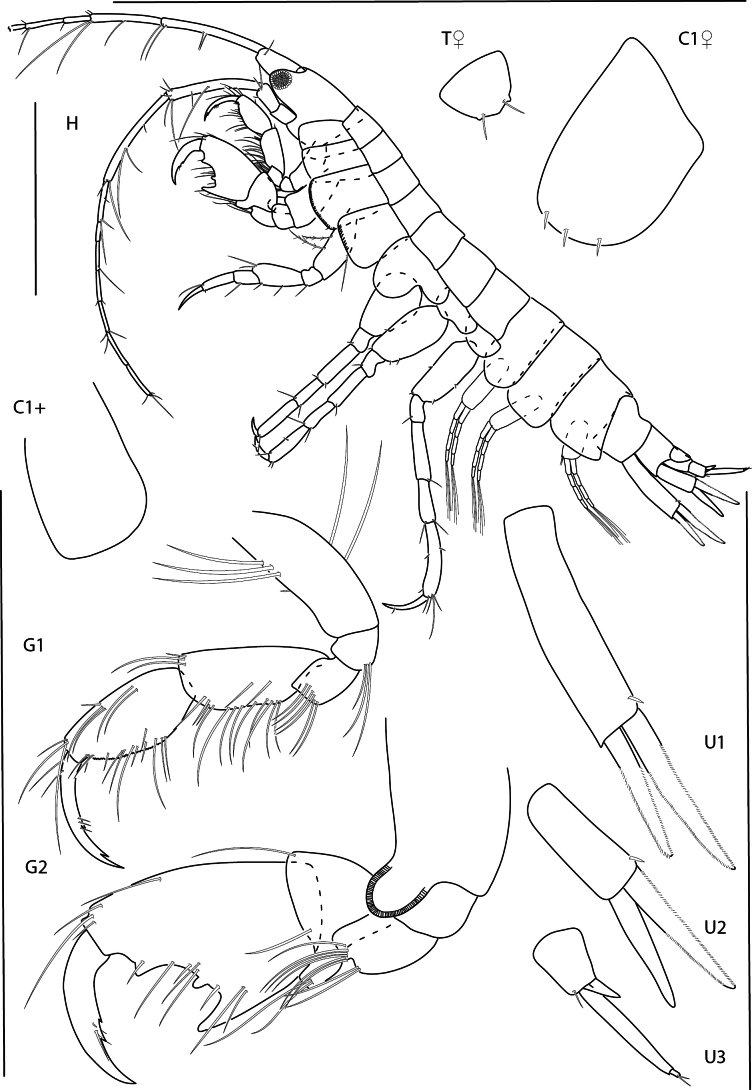
*Photisprobolion* sp. nov., male holotype, 1.4 mm, habitus, coxa 1, gnathopod 1 medial, gnathopod 2 medial, uropod 1, uropod 2, uropod 3; female paratype, 1.7 mm, telson, coxa 1. Scale bars: 0.5 mm.

**Figure 22.**
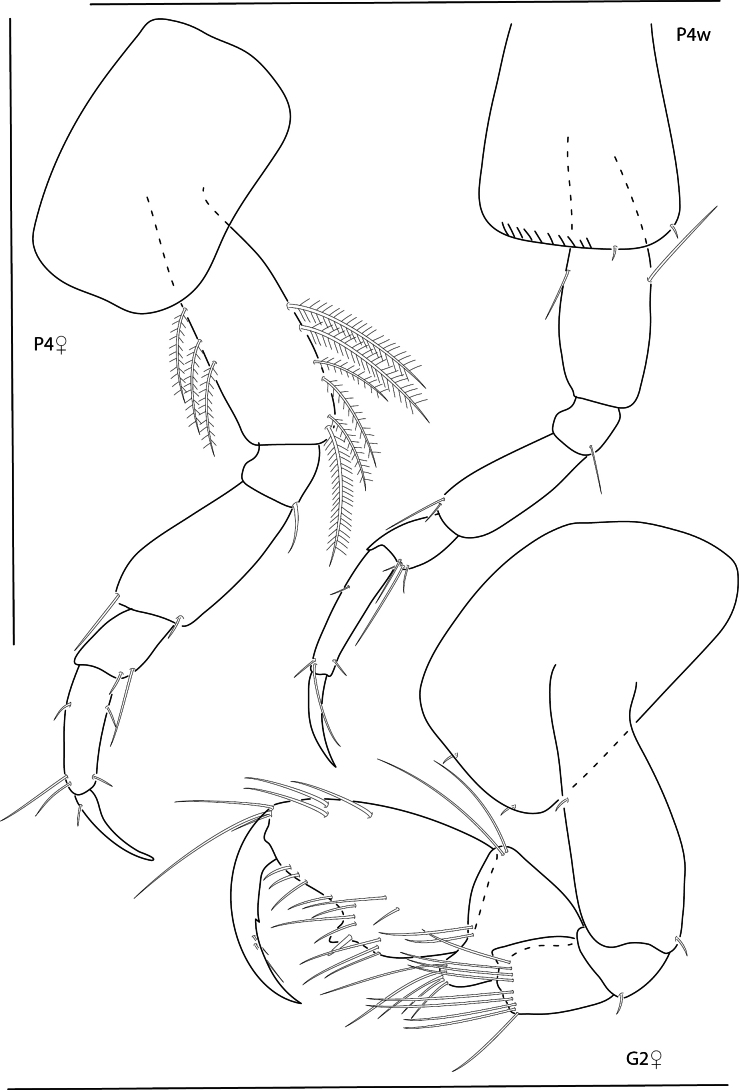
*Photisprobolion* sp. nov., female paratype, 1.7 mm, pereopod 4, gnathopod 2 medial; male paratype “w”, 1.6 mm, pereopod 4. Scale bars: 0.5 mm.

**Figure 23.**
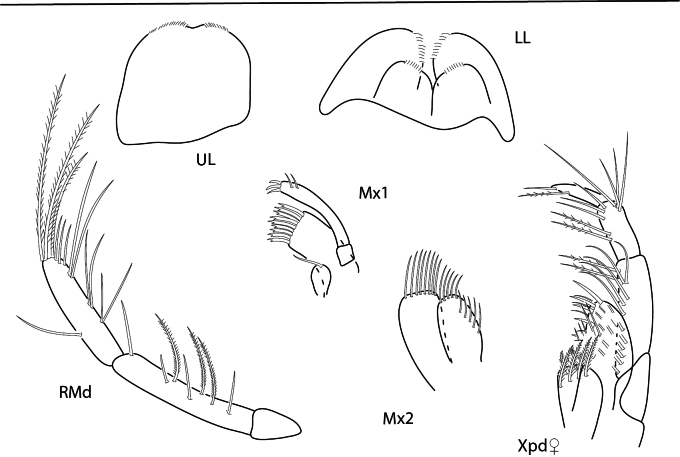
*Photisprobolion* sp. nov., male holotype, 1.4 mm, upper lip, lower lip, right mandible, maxilla 1, maxilla 2; female paratype, 1.7 mm, maxilliped. Scale bars: 0.5 mm.

Figs 21 , 22 , 23 , 32G

Photis sp. D: LeCroy 2000: 158, fig. 193.

###### Type locality.

Bocas del Toro, Panama: Crawl Caye, 9.2502°N, 82.1318°W, depth 5–13 m, among coral rubble.

###### Material examined.

***Holotype***: Panama • 1 ♂, 1.4 mm; Bocas del Toro, Crawl Caye; 9.2502°N, 82.1318°W; depth 10–13 m, among coral rubble; 29 June 2023; K.N. White leg; USNM 1743967. ***Paratypes***: Panama • 1 ♂, 1.6 mm; same station data as for preceding; USNM 1743968 • 1 ♀, 1.68 mm; same station data as for preceding; USNM 1743969. ***Other material***: Panama • 5 ♂, 2 ♀; same station data as for preceding; USNM 1743971 • 2 ♂, 4 ♀; Bocas del Toro, Crawl Caye; 9.2502°N, 82.1318°W; depth 5 m, among coral rubble; 29 June 2023; K.N. White leg; USNM 1743970.

###### Diagnosis.

Male coxa 1 anteroventral angle rounded, without setae. Male gnathopod 2 basis with large anterodistal lobe, lined with stridulating ridges; propodus process at palmar angle long, slender, curved. Male coxae 3 and 4 with stridulating ridges. Uropods 1–3 rami without marginal robust setae. Uropod 3 inner ramus lanceolate, inner ramus 0.2 × length of outer ramus, outer ramus, bi-articulate, article 2 with at least one apical seta.

###### Description.

**Male** (holotype, 1.7 mm). *Head*. Eyes round, circle of dark ommatidia surrounded by light ommatidia, touching anterior margin of ocular lobe. Antenna 1 broken; peduncle article 2 2.3 × length of article 1. Antenna 2 flagellum 7-articulate. Maxilliped inner plate apical margin with two rows of plumose setae; outer plate with four robust setae, lined with submarginal setae; palp 4-articulate. Lower lip inner and outer lobes rounded, lined with fine setae. Maxilla 1 inner plate small with one apical seta; outer plate with eight bifurcate robust setae; palp bi-articulate with three apical robust setae and two marginal setae. Maxilla 2 outer plate with eight apical setae; inner plate with five apical and five marginal setae. Mandibles similar, incisors dentate; palp tri-articulate, articles 2 and 3 setose, with some plumose setae. Upper lip rounded with indentation, apically setose on either side of indentation.

***Pereon.*** Coxae sparsely setose, setae short; coxae 1–4 longer than wide; coxae 3–4 with stridulating ridges. Gnathopod 1 subchelate; basis unexpanded, margins with tufts of long setae; carpus 0.1× length of propodus; propodus palm serrate, lacking robust setae; dactylus minutely serrate. Gnathopod 2 basis with large anterodistal lobe, lined with stridulating ridges; propodus process at palmar angle long, slender, curved. Pereopod 3 merus minutely expanded anteriorly. Pereopod 4 anterodistal margin of carpus with pointed projection, with few setae. Pereopods 5–7 bases rounded, narrowing sequentially. Pereopod 7 significantly longer than pereopods 5 and 6.

***Pleon.*** Epimeron 3 posteroventral corner subquadrate without notch. Uropod 1 peduncle with one distal robust seta, 1.3 × length of inner ramus; inner ramus 1.3 × length of outer ramus, rami lined with minute setules, lacking robust setae. Uropod 2 peduncle 0.8 × length of inner ramus, with one distal robust seta; inner ramus 1.2 × length of outer ramus, lined with minute setules, lacking robust setae. Uropod 3 peduncle 2.0 × length of inner ramus with one apical seta; inner ramus lanceolate, 0.2 × length of outer ramus, bare; outer ramus bi-articulate, article 2 with two apical setae.

**Male** (paratype, 1.6 mm). *Head.* Antenna 1 shorter than antenna 2, distal margins with sparse, long setae, flagellum tri-articulate, terminal article minute. Antenna 2 flagellum 4-articulate, terminal article minute. *Pleon.* Telson entire, apex subtriangular, with two setae, lateral margins with subacute points.

**Female** (paratype, 1.7 mm). Similar in all aspects to the male with the exception of the following: antenna 1 5-articulate; antenna 2 8-articulate; coxae without stridulating ridges; coxa 1 ventral margin with few short setae; gnathopod 2 basis anterodistal margin unexpanded, without lobe; propodus with reduced distal thumb, lacking triangular process near insertion of dactylus; pereopod 4 basis anterior and posterior margins with long plumose setae, anterodistal margin of carpus without pointed projection.

###### Etymology.

After the Latin *probolos*, meaning any projecting or jutting object or prominence and referring to large anterodistal lobe on the basis of the gnathopod 2 of males of this species.

###### Distribution.

U.S.A.: Florida: Biscayne Bay, Florida Bay (LeCroy 2000); Panama: Bocas del Toro (present study).

###### Ecology and remarks.

This species occurs among coral rubble at depths of 1.6–13 m. Panamanian specimens closely resemble *Photis* sp. D LeCroy, 2000 with the exception of male gnathopod 2 propodus having a small, rounded process near palmar angle and a smaller thumb at palmar angle, which is most likely due to the small size of the Panamanian specimens. *Photisprobolion* sp. nov. most closely resembles *Photislongicaudata* (Bate & Westwood, 1862) and *Photisbronca*Jung et al., 2019 in sharing the rounded anteroventral angle on coxa 1 and the large anterodistal lobe with stridulating ridges on gnathopod 2 basis. The new species differs from *P.longicaudata* in having stridulating ridges on male coxa 4 and in lacking robust setae on uropods 1 and 2 outer rami. The new species differs from *P.bronca* in lacking setae on coxa 1 and in having uropod 3 peduncle shorter than the inner ramus (vs peduncle longer than inner ramus). *Photisprobolion* sp. nov. can be easily distinguished from all other described Photis species in having a large anterodistal lobe lined with stridulating ridges on the male gnathopod 2 basis, the general shape of the gnathopod 2 propodus, stridulating ridges on male coxae 3 and 4, and in lacking marginal robust setae on uropods 1–3 rami. Live specimens are white, sometimes with faint brown stripes and pink markings, especially on the antennae.

##### ﻿ Photis melanica

Taxon classificationAnimaliaAmphipodaPhotidae

McKinney, 1980

137F61DC-8853-5679-A90D-CBB928E8E7E0

Figs 24 , 32H

Photis melanicus McKinney, 1980: 57–61, fig. 1.
Photis melanica : LeCroy 2000: 154, fig. 187; LeCroy et al. 2009: 941–972.
Photis sp. B: McKinney 1977: 112, fig. 19.

###### Material examined.

Panama • 1.7 mm • 1 ♀; Bocas del Toro, Laboratory Dock; 9.4159°N, 82.2489°W; depth 14 m, in light trap; 8 Aug 2005; T.A. Haney leg.; GCRL 6671.

###### Diagnosis.

Ocular lobe distal margin subacute. Coxae 1–4 ventral margins lined with long setae. Coxa 1 anteroventral margin evenly rounded. Female gnathopod 2 propodus palmar margin lacking process. Pereopod 3 merus with long plumose setae. Pereopod 5 basis bare. Pereopods 6 and 7 propodus with few setae shorter than dactylus; dactylus with accessory claw. Uropod 1 inner ramus 1.2 × length of outer ramus. Uropod 3 peduncle with plumose distoventral seta; inner ramus 0.2 × length of outer ramus, apical margin with setae.

###### Distribution.

U.S.A.: Florida Bay, Tampa Bay, Florida (LeCroy 2000), South of Galveston, Texas (McKinney 1977, 1980), Gulf of Mexico (LeCroy et al. 2009); Venezuela: exact location unspecified (Martín and Diaz 2003; LeCroy et al. 2009). Panama: Bocas del Toro (present study).

**Figure 24.**
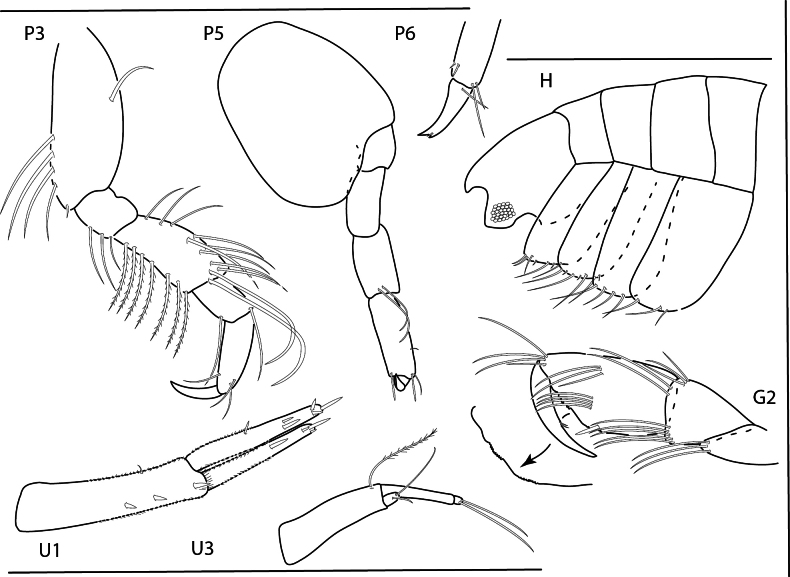
*Photismelanica*, female, 1.7 mm, pereopod 3, pereopod 5, pereopod 6 dactylus, head and coxae 1-4; uropod 1, uropod 3, gnathopod 2 medial. Scale bars: 0.5 mm.

###### Ecology and remarks.

This species was collected with light traps at a depth of 14 m. Panamanian specimens agree closely with previous descriptions. Ethanol-preserved specimens retained faint brown coloration on most of body.

##### ﻿Genus Posophotis

Taxon classificationAnimaliaAmphipodaPhotidae

Barnard, 1979

3F364B09-BEA3-5747-993C-0660E65DDEA8

###### Diagnosis.

Antenna 1 accessory flagellum 2- or 3-articulate (possibly with tiny additional article). Head cephalic lobe subacute. Coxae elongate, large, and overlapping. Dactylus of maxilliped short, stubby, setose apically. Gnathopod 1 small, weakly subchelate, carpus slightly longer than propodus. Gnathopod 2 broad, subchelate, posterior margin of propodus broadly lobate, anterior margin of propodus slightly longer than posterior margin, palm oblique, sculptured. Uropod 3 peduncle elongate, rami styliform, slightly shorter than peduncle, one-articulate.

##### ﻿ Posophotis seri

Taxon classificationAnimaliaAmphipodaPhotidae

Barnard, 1979

89F97D3E-555D-50CB-8BD3-0BB307D09EAA

Figs 25 , 33A

Posophotis seri Barnard, 1979: 31–34, figs 11, 12.

###### Material examined.

Panama • 2.3–4.7 mm • 2 ♀; Bocas del Toro, Bocas del Drago, 9.4180°N, 82.3375°W, depth 2.4 m, among red algae; 9 Aug 2021; K.N. White leg.; USNM 1743972.

###### Diagnosis.

Eye round. Gnathopod 1 propodus without projections. Female gnathopod 2 propodus with mid-palmar excavation, large robust seta at palmar angle; dactylus longer than palm. Uropod 3 peduncle elongate, rami styliform, lined with minute setae, each ramus with one marginal robust seta.

**Figure 25.**
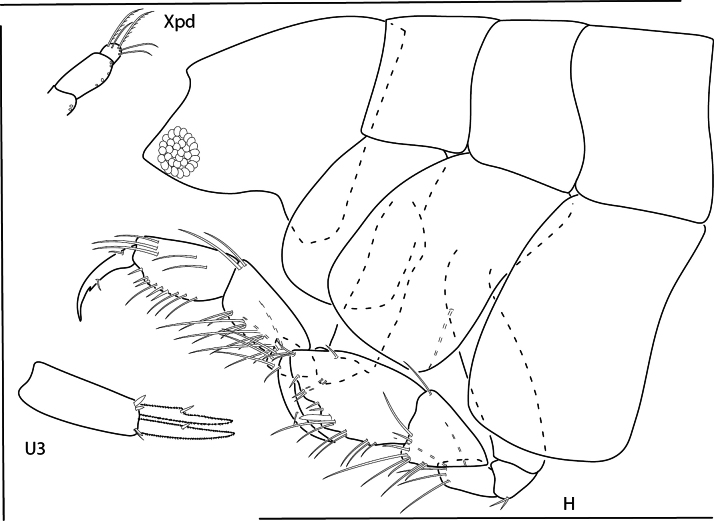
*Posophotisseri*, female, 4.7 mm, maxilliped palp (in part), habitus (in part), uropod 3. Scale bars: 0.5 mm.

###### Distribution.

Mexico: Puerto Peñasco (Barnard 1979); Ecuador: Galapagos Islands (Barnard 1979); Panama: unspecified (Barnard 1979), Bocas del Toro (present study).

###### Ecology and remarks.

This species occurs among red algae at a depth of 2.4 m. Panamanian specimens closely resemble specimens described in Barnard, 1979. Ethanol-preserved specimens retained brown coloration on pereon.

#### ﻿Family Podoceridae Leach, 1814

##### ﻿Genus Podocerus

Taxon classificationAnimaliaAmphipodaPodoceridae

Leach, 1814

85B04EC6-6A02-5A42-AAD0-21D6F1AD57E1

###### Diagnosis.

Body often with dorsal elevations or carinate, pereon segments 2 and 3 fused. Antenna 2 longer than antenna 1, stout. Head not globular, buccal mass similar in size to head. Coxae discontiguous. Urosomites separate, urosomite 1 elongate. Uropods 1–3 dissimilar in structure; uropod 3 greatly reduced.

##### ﻿ Podocerus offucia sp. nov.

Taxon classificationAnimaliaAmphipodaPodoceridae

0525432E-5411-5290-A814-C02C34AEB37D

https://zoobank.org/B828F2DD-0E9C-49EE-A0E4-580D7F3D6E9E

**Figure 26.**
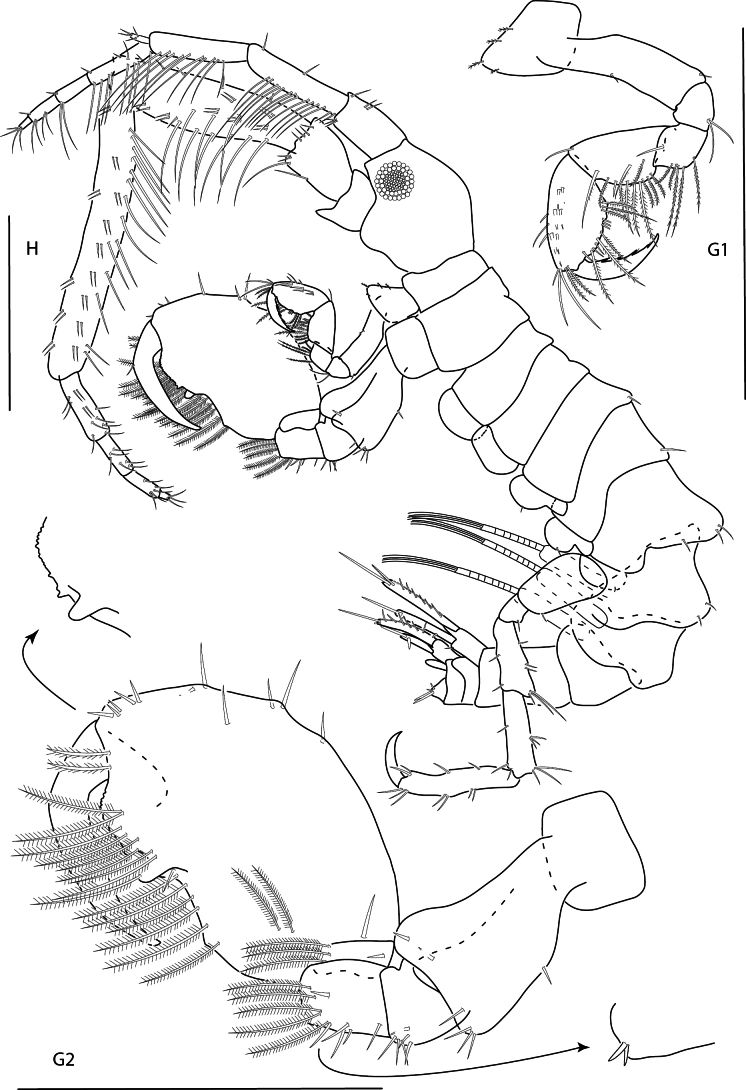
*Podocerusoffucia* sp. nov., male holotype, 2.8 mm, habitus, gnathopod 2 medial, gnathopod 2 medial. Scale bars: 0.5 mm.

**Figure 27.**
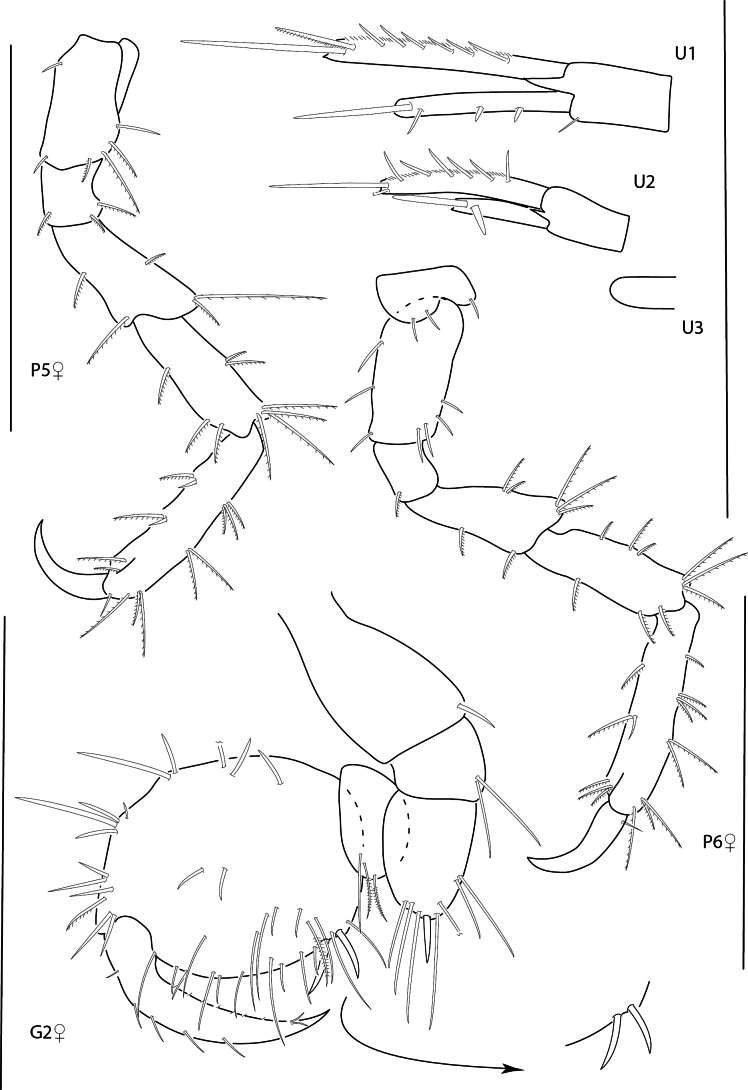
*Podocerusoffucia* sp. nov., female paratype, 2.8 mm, pereopod 5, gnathopod 2 medial, pereopod 6; male holotype, 2.8 mm, uropod 1, uropod 2, uropod 3. Scale bars: 0.5 mm.

**Figure 28.**
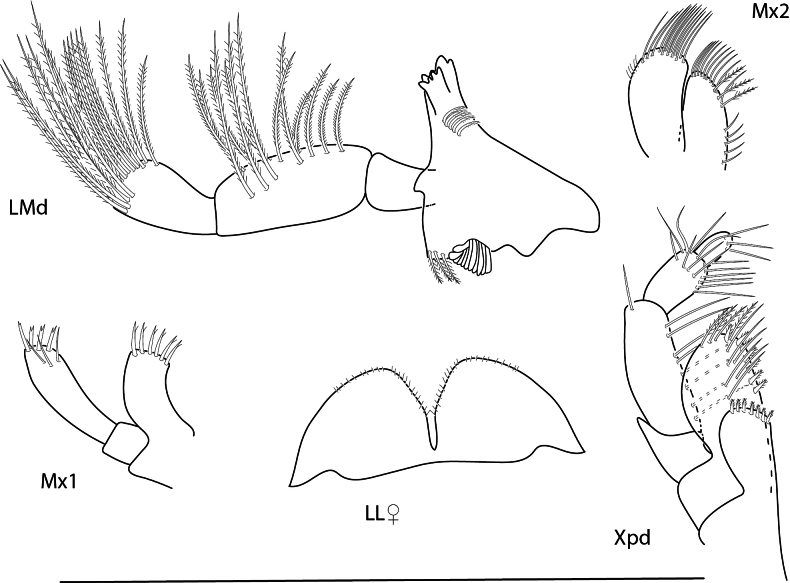
*Podocerusoffucia* sp. nov., male holotype, 2.8 mm, left mandible, maxilla 1, maxilla 2, maxilliped; female paratype, 2.8 mm, lower lip. Scale bars: 0.5 mm.

Figs 26 , 27 , 28 , 33B

###### Type locality.

Bocas del Toro, Panama: Swan Cay, 9.4536°N, 82.3000°W, among coral rubble.

###### Material examined.

***Holotype***: Panama • 2.8 mm • 1 ♂; Bocas del Toro, Panama: Swan Cay, 9.4536°N, 82.3000°W, depth 1–3 m; among coral rubble; 27 June 2023; K.N. White leg; USNM 1743973. ***Paratype***: Panama • 2.8 mm • 1 ♀; station data same as holotype; USNM 1743974.

###### Diagnosis.

Pereon segments 5–7 dorsally setose. Male antenna 1 flagellum 4-articulate; accessory flagellum uni-articulate. Coxa 1 with four marginal setae. Gnathopod 1 basis slender. Male gnathopod 2 propodus palm serrate with one large, rounded projection, posteriorly concave. Pereopods 3–7 propodus with posterodistal notch. Uropods 1 and 2 each with distoventral interramal spine.

###### Description.

**Male** (holotype, 2.8 mm). ***Head*.** Eyes round, bulging. Antenna 1 densely setose, flagellar article 1 shorter than flagellar article 2; accessory flagellum uni-articulate. Antenna 2 densely setose; flagellum 4-articulate. Maxilliped inner plate with seven plumose apical setae, outer plate lined with ten marginal robust setae and many facial setae, palp 4-articulate. Lower lip missing. Maxilla 1 inner plate absent; outer plate with six apical robust setae; palp bi-articulate, apical margin with four bifurcate setae and three slender setae. Maxilla 2 inner and outer plate apical margins lined with setae; outer plate with two rows. Mandibular palp tri-articulate, lined with plumose setae. Upper lip missing.

***Pereon*.** Pereon segments with posterodorsal processes. Pereonites 5–8 with dorsal robust setae. Coxae sparsely setose, discontiguous. Coxa 1 rhomboidal. Gnathopod 1 subchelate; basis slender; ischium posterior margin with two long setae; merus posterior margin with long plumose setae; carpus longer than wide, posterior margin lined with plumose setae, with two plumose facial setae; propodus subequal in length with carpus, posterior margin with many simple and plumose setae, anterolateral margin with facial setae; dactylus inner margin serrate. Gnathopod 2 subchelate, much larger than gnathopod 1; basis expanded anteriorly into rounded process, posterior margin with short setae; ischium posteriodistal margin with sparse short setae; merus ventral margin lined with long slender plumose setae and two short, stout setae; carpus reduced, ventral margin lined with long slender plumose setae; propodus palm serrate with one large, rounded projection, posteriorly concave; dactylus closing on concavity. Pereopods 3–6 missing. Pereopod 7 basis ovate; ischium short; merus posteriorly expanded; propodus with posterodistal notch.

***Pleon*.** Epimera 1–3 with posterodorsal processes; posteroventral margins smooth, rounded. Uropod 1 1.4 × length of uropod 2; peduncle with distoventral interramal spine, apical margin with one slender seta; inner ramus 2.3 × length of peduncle, outer margin lined with robust setae and fine setules, apical margin with one long seta; outer ramus outer margin with few robust setae, apical margin with one long seta. Uropod 2 peduncle with distoventral interramal spine, apical margin with one slender seta; inner ramus 2.9 × length of peduncle, outer margin lined with robust setae and fine setules, apical margin with one long seta; outer ramus outer margin with one robust seta, apical margin with one long seta. Uropod 3 vestigial, rounded. Telson dorsally produced, with two long and two short setae.

**Female** (paratype, 2.8 mm). Similar in all aspects to the male with the exception of the following: Antenna 1 flagellum tri-articulate. Lower lip outer lobes rounded, setose; mandibular lobes acutely pointed. Gnathopod 2 basis stout, without rounded process, posterodistal margin with one seta; merus rounded posteriorly, with distal robust seta and several slender setae; carpus subtriangular; propodus enlarged, rounded, palmar margin smooth, moderately setose, with two large setae; dactylus inner margin with short setae. Pereopods 5 and 6 anterior and posterior margins of most articles with plumose setae; propodus posterodistal margin with notch.

###### Etymology.

After the Latin *offucia*, meaning paint or wash for face and referring to dark pigment on the head of this species.

###### Distribution.

Panama: Bocas del Toro (present study).

###### Ecology and remarks.

These amphipods occur among coral rubble, occurring with other *Podocerus* species at depths of 1–3 m. *Podocerusoffucia* sp. nov. is most similar to *Podoceruslazowaemi* Baldinger & Gable, 1994 in sharing the following characters: male antenna 1 flagellum 4-articulate, accessory flagellum uni-articulate; gnathopod 1 basis slender; and uropods 1–2 each with distoventral interramal spine. The new species differs in the following characters: antenna 2 flagellum 4-articulate (vs 5-articulate), coxae 1 with four setae (vs 1 robust seta), coxa 2 bare (vs 1 robust seta), pereopods 3–7 propodus with posterodistal notch (vs lacking). *Podocerusoffucia* sp. nov. is also similar to *Podoceruskleidus* Thomas & Barnard, 1992 in the presence of interramal spines on uropod 1 and 2 but differs in the following characters: antenna 1 flagellum 3 or 4-articulate (vs 6-articulate), coxa 1 entire (vs cleft), and four robust setae on telson (vs 9 robust setae). *Podocerusoffucia* sp. nov. can be easily distinguished from all remaining described *Podocerus* species based on having dorsal setae only on pereon segments 5–7, the shape of male gnathopod 2 propodus, and having uropods 1 and 2 each with distoventral interramal spine. Ethanol-preserved specimens of the new species retained purple-brown coloration on the head with faint coloration on the pereon.

##### ﻿ Podocerus fissipes

Taxon classificationAnimaliaAmphipodaPodoceridae

Serejo, 1995

55A77FA2-18A0-5AF2-9994-36110BB6FF09

Figs 29 , 33C

Podocerus fissipes Serejo, 1995[1996]: 49–55, figs 1–3; Baldinger and Gable 2002: 11–19, figs 6–12; LeCroy 2011: 702, fig. 560.

###### Material examined.

Panama • 3–5.1 mm • 2 ♂, 5 ♀; Bocas del Toro, Hospital Bight, 9.3044°N, 82.1316°W, depth 1.5 m, among coral rubble; 7 Aug 2005; S. LeCroy leg.; GCRL 6672 • 3 ♂, 8 ♀, 2 juveniles; Bocas del Toro, Hospital Point, 9.3333°N, 82.2185°W, depth 11 m, from buoy scrapings; 26 June 2023; K.N. White leg.; USNM 1743975 • 9 ♂, 7 ♀, 1 juvenile; Bocas del Toro, Crawl Caye, 9.2475°N, 82.1290°W, depth 0–1 m, from buoy scrapings; 28 June 2023; K.N. White leg.; USNM 1743976.

###### Diagnosis.

Maxilla 2 inner and outer plates each with two rows of apical setae. Gnathopod 2 merus without robust setae; propodus with two robust setae at palmar angle. Uropods 1 and 2 without interramal spines. Telson apex truncate with two long and two short setae.

**Figure 29.**
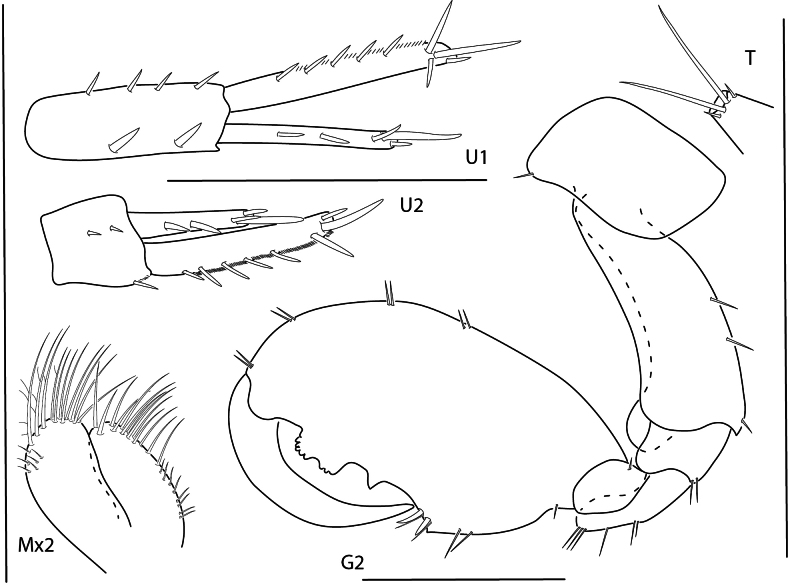
*Podocerusfissipes*, male, 4.6 mm, uropod 1, uropod 2, maxilla 2, gnathopod 2 lateral, telson apex. Scale bars: 0.5 mm.

###### Distribution.

Brazil: Prainha in Arraial do Cabo, Rio de Janeiro; Santo Aleixo Island, Serinhaém, Pernambuco (Serejo 1995); British Virgin Islands: Guana (Baldinger and Gable 2002); U.S.A.: Biscayne Bay, Florida (LeCroy 2011); Bocas del Toro (present study).

###### Ecology and remarks.

Panamanian specimens agree with previous descriptions of *Podocerusfissipes* with the following exceptions: variation in dorsal carinae; gnathopod 2 with two robust setae (vs one robust seta in Serejo 1995 and LeCroy 2011). Baldinger and Gable (2002), however, also describe two robust setae on the gnathopod 2 propodus. The variation may be due to the size difference (Panama = 4.6 mm, B.V.I. = 3.0 mm, Brazil = 2.3 mm) or due to regional variation. Live specimens have orange coloration lining pereonites and are covered with orange speckles.

##### ﻿ Podocerus jareckii

Taxon classificationAnimaliaAmphipodaPodoceridae

Baldinger & Gable, 2002

E6C6D5F4-7402-52A4-AB93-A50EC8FC6531

Figs 30 , 33D

Podocerus jareckii Baldinger & Gable, 2002: 3–11, figs 1–5.

###### Material examined.

Panama • 2.4 mm • 1 ♂, 1 ♀; Bocas del Toro, Swan Cay, 9.4536°N, 82.3000°W, among coral rubble; 27 June 2023; K.N. White leg; USNM 1743977.

###### Diagnosis.

Maxilla 2 inner and outer plates each with one row of apical setae. Gnathopod 2 propodus with proximal robust seta, dactylus bent at angle. Uropod 1 interramal spine present. Uropod 2 interramal spine absent.

**Figure 30.**
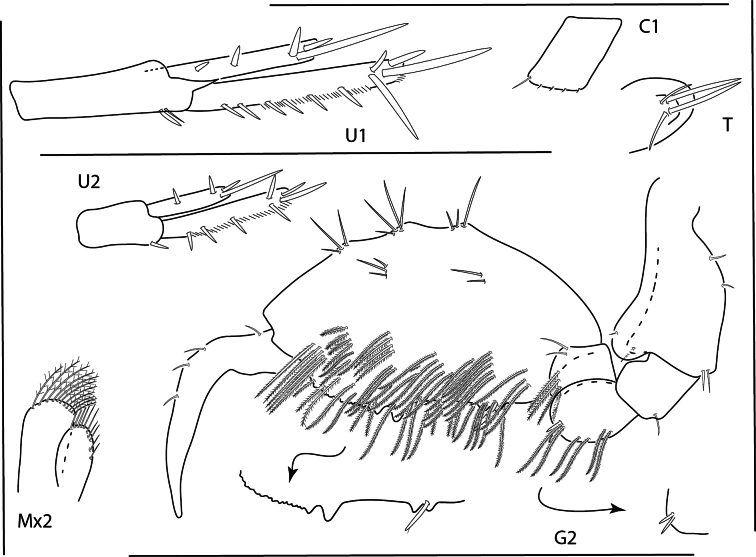
*Podocerusjareckii*, male, 2.4 mm, uropod 1, uropod 2, maxilla 2, coxa 1, telson, gnathopod 2 medial. Scale bars: 0.5 mm.

###### Distribution.

British Virgin Islands: Guana Island (Baldinger and Gable 2002); Panama: Bocas del Toro (present study).

###### Ecology and remarks.

This species occurs among coral rubble at a depth of 2.3–2.4 m. Panamanian specimens closely resemble the original description with the following exceptions: male dorsal robust setae starting on pereonite 5–7 (vs pereonite 2–7); gnathopod 2 merus with two robust setae (vs 1); telson with four apical setae (vs 5). Live specimens are orange, purple-red, and white in color.

### ﻿Identification Key to the Caribbean Caprellidira of Panama

**Table:** 

1	Head anteroventral margin entire, rounded; pereonite 1 fused to head; body skeletal, segments tubular; gills not exceeding 3 pairs; oostegites not exceeding 2 pairs; abdomen vestigial (Fig. 1)	**2**
–	Head anteroventral margin recessed, excavate; head free from pereonite 1; body compressed or flattened; gills exceeding 3 pairs; oostegites exceeding 2 pairs; abdomen well developed (Figs 26, 27)	**4**
2	Body without dorsal projections; male pereonite 2 with sharp projection on anteroventral margin; male gnathopod 2 basis posterior margin with proximal bump (Fig. 3)	***Paracaprellapusilla***
–	Body with dorsal projections; male pereonite 2 without projection on anteroventral margin; male gnathopod 2 basis posterior margin smooth (Fig. 1)	**3**
3	Pereopods 3 and 4 are bi-articulate (Fig. 2)	**Deutellacf.pseudoincerta**
–	Pereopods 3 and 4 are uni-articulate (Fig. 1)	***Deutellacaribensis***
4	Antenna 2 distinctly longer than antenna 1, antenna 1 not reaching past antenna 2 peduncle (Fig. 26); urosome segment 1 longer than deep, distinctly longer than segment 2 (Fig. 26)	**5**
–	Antenna 2 slightly longer than antenna 1, antenna 1 usually reaching past antenna 2 peduncle (Fig. 5); urosome segment 1 at least as deep as long, not distinctly longer than segment 2 (Fig. 9)	**7**
5	Maxilla 2 inner and outer plates each with one row of apical setae (Fig. 28); gnathopod 2 merus with robust setae; uropod 1 interramal spine present (Fig. 27)	**6**
–	Maxilla 2 inner and outer plates each with two rows of apical setae; gnathopod 2 merus without robust setae; uropod 1 interramal spine absent (Fig. 29)	***Podocerusfissipes***
6	Male gnathopod 2 propodus posterior margin straight; dactylus bent at angle; uropod 2 without interramal spine (Fig. 30)	***Podocerusjareckii***
–	Male gnathopod 2 propodus posterior margin concave; dactylus evenly curved (Fig. 26); uropod 2 with interramal spine (Fig. 27)	***Podocerusoffucia* sp. nov.**
7	Gnathopod 1 subequal or larger than gnathopod 2, carpus longer than or subequal to propodus (Fig. 9)	**8**
–	Gnathopod 1 smaller than gnathopod 2, carpus shorter than or subequal to propodus (Fig. 8)	**9**
8	Male coxa 1 subquadrate, not significantly larger than coxa 2; male gnathopod 1 chelate, carpus and propodus fused, dactylus with elongate tooth along posteroproximal margin; male pereopod 5 basis length 1.4 × width; telson apex convex (Fig. 12)	***Varohiostopianus***
–	Male coxa 1 subovate, significantly larger than coxa 2; male gnathopod 1 carpochelate, 7-articulate, dactylus lacking elongate tooth along posteroproximal margin; male pereopod 5 basis length 2.6 × width; telson apex concave (Figs 9, 10)	***Konatopustridens* sp. nov.**
9	Coxae not overlapping (Fig. 8); pereopods 3 and 4 distinctly expanded (Figs 4, 8); uropod 3 uniramous (Fig. 8) or lacking rami (Fig. 4)	**10**
–	Coxae overlapping; pereopods 3 and 4 bases slightly or not expanded; uropod 3 biramous (one ramus may be much smaller than other) (Fig. 21)	**14**
10	Gnathopod 1 simple; gnathopod 2 propodus palm lined with stout setae; uropod 2 absent; uropod 3 lacking rami (Fig. 4)	***Caribboecetesintermedius***
–	Gnathopod 1 subchelate; gnathopod 2 propodus palm not lined with stout setae; uropod 2 present; uropod 3 uniramous or biramous (Fig. 8)	**11**
11	Uropod 1 inner ramus subequal in length with outer ramus; pereopod 5 not geniculate; uropod 2 biramous; telson entire (Fig. 8)	***Ericthoniusbrasiliensis***
–	Uropod 1 inner ramus shorter than outer ramus; pereopod 5 geniculate (Fig. 5); uropod 2 uniramous (Fig. 7); telson cleft (Figs 5, 7)	**12**
12	Female pereopod 3 basis at right angle posteriorly; telson partially cleft (Fig. 7)	***Cerapusthomasi***
–	Female pereopod 3 basis not at right angle posteriorly; telson entirely cleft (Fig. 5)	**13**
13	Antennae 1 and 2 with > 3 flagellar segments; uropod 1 inner ramus with apical robust seta, distal margin of seta narrowing unevenly (Fig. 5)	***Cerapusbenthophilus***
–	Antennae 1 and 2 with 3 flagellar segments; uropod 1 inner ramus with apical robust seta, distal margin of seta narrowing evenly (Fig. 6)	***Cerapusslayeri***
14	Male antenna 2 flagellum dorsoventrally flattened; female gnathopod 1 merus and carpus with ventral setae, all setae plumose; female gnathopod 2 propodus anterior margin with dense rows of setae; male uropod 3 rami subequal in length (Fig. 13)	***Audullachelifera***
–	Male antenna 2 flagellum not dorsoventrally flattened; female gnathopod 1 merus and carpus with or without ventral setae, if present none or few setae plumose; female gnathopod 2 propodus anterior margin without dense rows of setae; male uropod 3 rami unequal in length (Fig. 6)	**15**
15	Antenna 1, accessory flagellum vestigial or absent; uropod 3, inner ramus minute (Fig. 21)	**16**
–	Antenna 1, accessory flagellum present, composed of > 1 article; uropod 3, inner ramus not minute (Fig. 6)	**17**
16	Eye touching margin of ocular lobe; pereopod 5 basis length 1.5 × width; uropods 1 and 2 outer ramus without marginal robust setae (Fig. 21)	***Photisprobolion* sp. nov.**
–	Eye not touching margin of ocular lobe; pereopod 5 basis length < 1.5 × width (Fig. 18); uropods 1 and 2 outer ramus with marginal robust setae (Fig. 19)	**18**
17	Coxa 1 anteroventral margin slightly produced with gap in marginal setae (Fig. 18); pereopods 6 and 7 propodus each with posterodistal cluster of setae surpassing length of dactylus (Fig. 19)	***Photisbulla* sp. nov.**
–	Coxa 1 anteroventral margin not produced without gap in marginal setae; pereopods 6 and 7 propodus posterodistal cluster of setae, if present, no longer than length of dactylus (Fig. 24)	**19**
18	Ocular lobe distal margin subacute; pereopod 3 merus posterior margin with several long, plumose setae; male pereopod 6 normal (based on literature); uropod 1 rami unequal in length; uropod 3 peduncle with plumose distoventral seta (Fig. 24)	***Photismelanica***
–	Ocular lobe distal margin rounded; pereopod 3 merus posterior margin without plumose setae; male pereopod 6 greatly enlarged; uropod 1 rami subequal in length; uropod 3 peduncle without plumose distoventral seta (Fig. 11)	***Photisbutalus* sp. nov.**
19	Head cephalic lobe subacute; coxa 2 length 1.5 × width (Fig. 25)	***Posophotisseri***
–	Head cephalic lobe rounded; coxa 2 length subequal to width (Fig. 14)	***Latigammaropsisatlantica***

**Figure 31.**
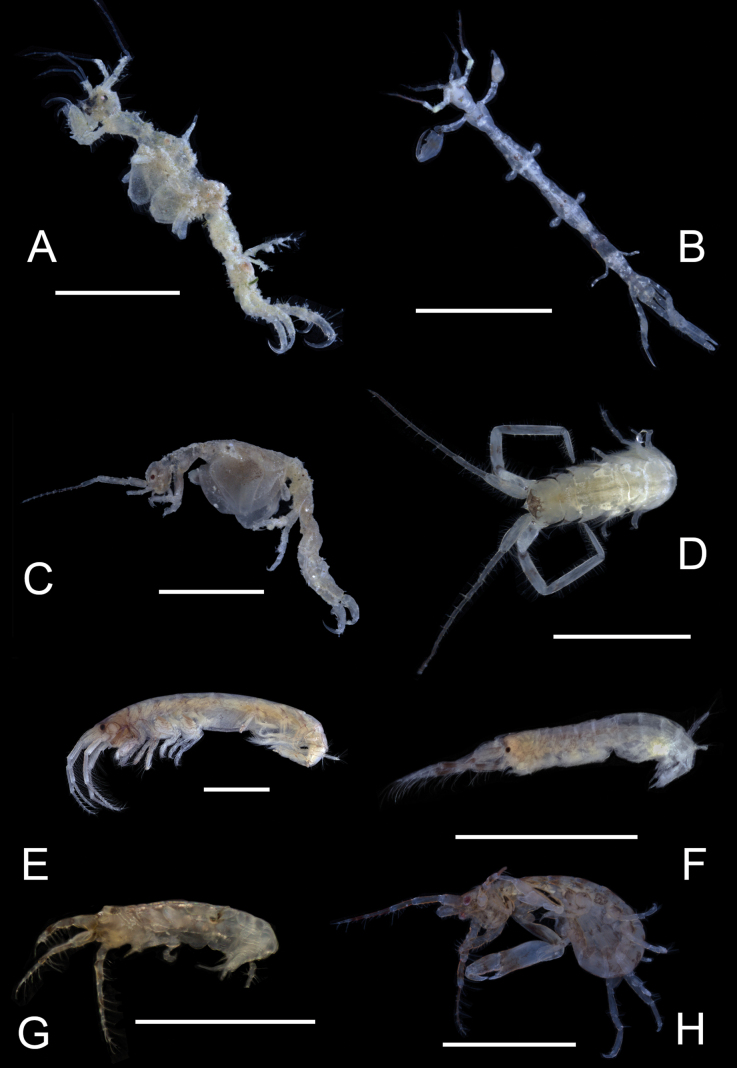
Photographs of live specimens unless noted. **A***Deutellacaribensis***B**Deutellacf.pseudoincerta**C***Paracaprellapusilla***D***Caribboecetesintermedius***E***Cerapusbenthophilus* (ethanol-preserved specimen) **F***Cerapusslayeri* (ethanol-preserved specimen) **G***Cerapusthomasi* (ethanol-preserved specimen) **H***Ericthoniusbrasiliensis*. Scale bars: 1.0 mm.

**Figure 32.**
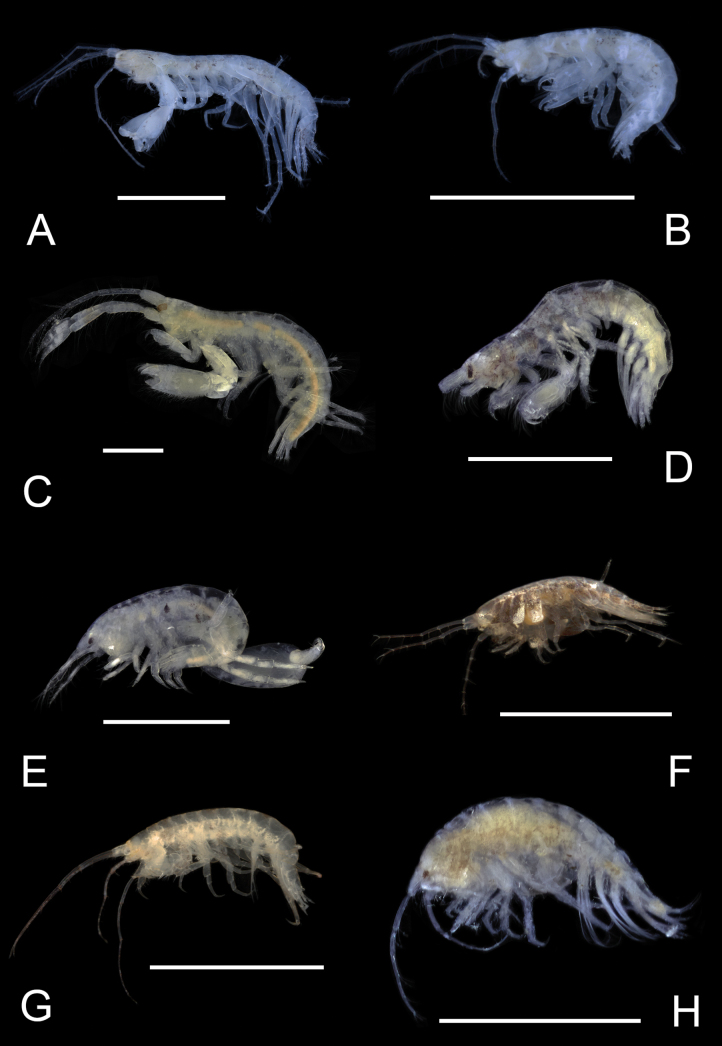
Photographs of live specimens unless noted. **A***Konatopustridens* sp. nov. **B***Variohostopianus***C***Audullachelifera* (ethanol-preserved specimen) **D***Latigammaropsisatlantica* (ethanol-preserved specimen) **E***Photisbutalus* sp. nov. (ethanol-preserved specimen) **F***Photisbulla* sp. nov. **G***Photisprobolion* sp. nov. **H***Photismelanica* (ethanol-preserved specimen). Scale bars: 1.0 mm.

**Figure 33.**
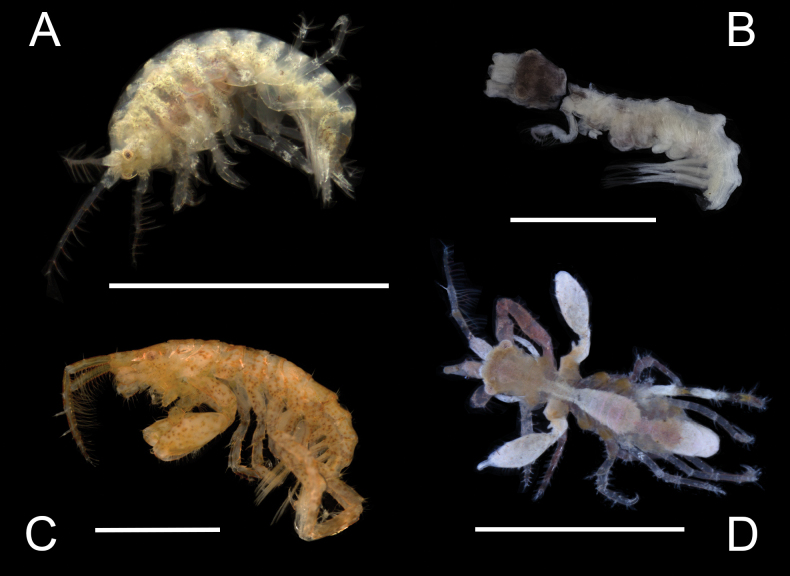
Photographs of live specimens unless noted. **A***Posophotisseri* (ethanol-preserved specimen) **B***Podocerusoffucia* sp. nov. (ethanol-preserved specimen) **C***Podocerusfissipes***D***Podocerusjareckii*. Scale bars: 1.0 mm.

## ﻿Discussion

This study describes five new species and includes a range extension for 15 caprellidiran amphipods to include the Caribbean waters of Panama. Five species have a distribution pattern including the Pacific and Caribbean (*Audullachelifera*, *Ericthoniusbrasiliensis*, *Latigammaropsisatlantica*, *Paracaprellapusilla*, *Posophotisseri*). These distribution patterns indicate that these five species were likely established before the isthmus of Panama closed, more than three million years ago. Examination of specimens has clarified that several characters vary and should not be used for species determination. Body spination should not be used as a diagnostic character among species of *Deutella* based on McCain (1968), Guerra-García et al. (2006), and Winfield and Guerra-García (2021). The number of antennae segments is also variable based on comparison of Panama material to original descriptions in the literature for Deutellacf.pseudoincerta, *Cerapusbenthophilus*, *Cerapusthomasi*, and *Latigammaropsisatlantica.* This study increases the known number of caprellidiran amphipods from Caribbean Panama from one to 21 species. The Caribbean Amphipoda of Panama identification key is available online (https://www.invertebase.org/portal/ident/key.php?clid=58&pid=4&dynclid=0&taxon=All+Species).

## Supplementary Material

XML Treatment for Deutella

XML Treatment for Deutella caribensis

XML Treatment for Deutella cf. pseudoincerta

XML Treatment for Paracaprella

XML Treatment for Paracaprella pusilla

XML Treatment for Caribboecetes

XML Treatment for Caribboecetes intermedius

XML Treatment for Cerapus

XML Treatment for Cerapus benthophilus

XML Treatment for Cerapus slayeri

XML Treatment for Cerapus thomasi

XML Treatment for Ericthonius

XML Treatment for Ericthonius brasiliensis

XML Treatment for Konatopus

XML Treatment for Konatopus tridens

XML Treatment for Varohios

XML Treatment for Varohios topianus

XML Treatment for Audulla

XML Treatment for Audulla chelifera

XML Treatment for Latigammaropsis

XML Treatment for Latigammaropsis atlantica

XML Treatment for Photis

XML Treatment for Photis butalus

XML Treatment for Photis bulla

XML Treatment for Photis probolion

XML Treatment for Photis melanica

XML Treatment for Posophotis

XML Treatment for Posophotis seri

XML Treatment for Podocerus

XML Treatment for Podocerus offucia

XML Treatment for Podocerus fissipes

XML Treatment for Podocerus jareckii

## ﻿Additional information

### Author ORCIDs

Sally J. Sir https://orcid.org/0000-0002-1270-1192

Kristine N. White https://orcid.org/0000-0002-5203-1656

### Data availability

All of the data that support the findings of this study are available in the main text or Supplementary Information.

## Supplementary materials

Supplementary material 1 Locality table Panama Caprellidira Data type xlsx https://binary.pensoft.net/file/1304915This dataset is made available under the Open Database License (http://opendatacommons.org/licenses/odbl/1.0/). The Open Database License (ODbL) is a license agreement intended to allow users to freely share, modify, and use this Dataset while maintaining this same freedom for others, provided that the original source and author(s) are credited.Sally J. Sir, Kristine N. White
